# Emergence of chirality and structural complexity in single crystals at the molecular and morphological levels

**DOI:** 10.1038/s41467-019-13925-5

**Published:** 2020-01-20

**Authors:** Maria Chiara di Gregorio, Linda J. W. Shimon, Vlad Brumfeld, Lothar Houben, Michal Lahav, Milko E. van der Boom

**Affiliations:** 1 Department of Organic Chemistry, Weizmann Institute of Science, 7610001 Rehovot, Israel; 2 Department of Chemical Research Support, Weizmann Institute of Science, 7610001 Rehovot, Israel

**Keywords:** Metal-organic frameworks, Crystal engineering

## Abstract

Naturally occurring single crystals having a multidomain morphology are a counterintuitive phenonomon: the macroscopic appearance is expected to follow the symmetry of the unit cell. Growing such crystals in the lab is a great challenge, especially from organic molecules. We achieve here uniform metallo-organic crystals that exhibit single crystallinity with apparently distinct domains and chirality. The chirality is present at both the molecular and macroscopic levels, although only achiral elements are used. “Yo-yo”-like structures having opposite helical handedness evolve from initially formed seemingly achiral cylinders. This non-polyhedral morphology coexists with a continuous coordination network forming homochiral channels. This work sheds light on the enigmatic aspects of fascinating crystallization processes occurring in biological mineralization. Our findings open up opportunities to generate new porous and hierarchical chiral materials.

## Abstract (web summary)

Single crystallinity combined with multidomain morphology is counterintuitive. Here, the authors achieve such a phenomenon in a metal-organic framework that also displays chirality at both the molecular and morphological levels.

## Introduction

Deciphering the complexity of crystal growth is one of the greatest open questions in chemistry since the seminal work of Louis Pasteur in 1848. The correlation between molecular packing and morphology combined with chirality is still poorly understood^1^. Likewise, it is difficult to form crystals that are uniform in size and shape. To control the crystal parameters, modulators, surfactants, and capping ligands have been used by others as additives^2–11^. Typically, crystals having multidomain morphologies are polycrystalline, whereas single crystals usually consist of one single unit. However, there are exceptions to this axiom. The growth of such crystals, which have no grain boundaries and which have a highly complex morphology, occurs in nature. An intriguing case is offered by the sea urchins’ spines^12,13^, which are single calcite crystals despite a curved and sponge-like appearance at the micrometer scale. Similarly, the irregular morphological boundaries of Foraminifera shells^14^ and the calcite elements constituting the complex structure of Coccoliths^15^ provide single crystal diffraction. The rules that govern the formation of crystals from organic molecules having similar contradictory structural features are presently unknown.

Chirality is an important additional aspect in this field, as chirality in conjunction with other much sought-after properties, such as porosity, enables applications related to enantioselective chemical transformations and separation^16–18^. Chiral 3D arrangements of assemblies of achiral molecules are well-known. Popplemeier and coworkers showed that even racemic mixtures can give chiral structures^19^ and Morris showed that bulk homochirality could be induced in metal organic frameworks (MOFs) in the right solvent systems^20^. The number of MOFs^21,22^ and zeolites^23,24^ with chiral crystallographic structures is increasing, the possibility to extend and combine chirality at different hierarchies remains unclear. To date, there have been just a few examples of MOFs with chiral surfaces^25,26^ or 3D twisted shapes^27^, where in the latter cases the chirality is induced by a template and is manifest at a morphological level. A fascinating prospect is to exploit chirality-associated effects at multiple size hierarchies to control the performance of new materials.

**Fig. 1:**
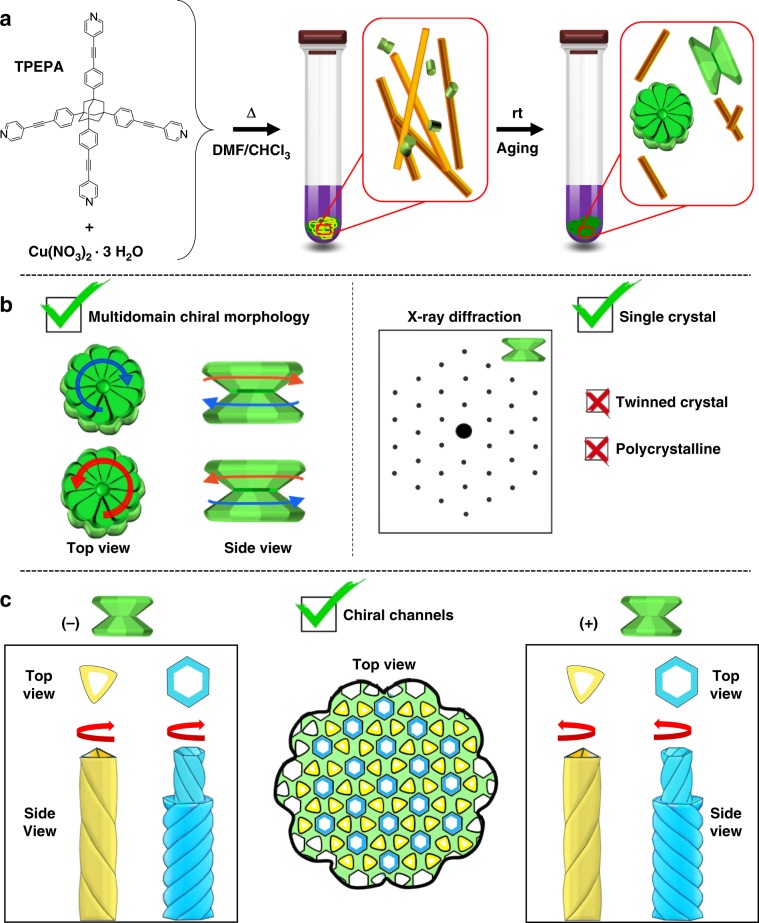
Single crystals with multidomain morphology, from achirality to multiple chirality. **a** solvothermal reaction of the achiral organic ligand (TPEPA) with copper(II) nitrate, followed by aging of the sample at room temperature. (**b**, left) The single crystals display chiral features at morphological levels: an off-set angle between the constituting disks and a spiral growth phase. (**b**, right) X-ray diffraction studies reveal the single crystallinity of the entire, morphological complex structure. **c** The crystal structure consists of continuous helicoidal channels. Each crystal is enantiopure whereas the bulk sample is racemic.

We report here the paradoxical growth of metallo-organic single crystals having a multidomain morphology (Fig. 1). Their macroscopic appearance clearly resembles a multifaceted and chiral structure. Strikingly, the seemingly separate domains are all parts of same single crystal. Spiral motifs span the entire crystal at both the morphological and crystallographic levels. The crystallization process involves a solvothermal reaction to generate morphologically achiral seeds, followed by the gradual development of homochirality. Coordination of achiral organic ligands to a metal cation leads to an asymmetric environment that directs the formation of continuous homochiral channels which are packed in a rare space group (*P*622).

A coordination network is the ideal vehicle to achieve such single crystal properties: the network continuity across the entire single crystal is characterized by definition by a perfect alignment of the unit cells. The mild crystal growth conditions might allow the material added from solution to be packed according to the structure of the dislocation planes and the chiral surface.

## Results

### Multidomain and chiral morphology

**Fig. 2:**
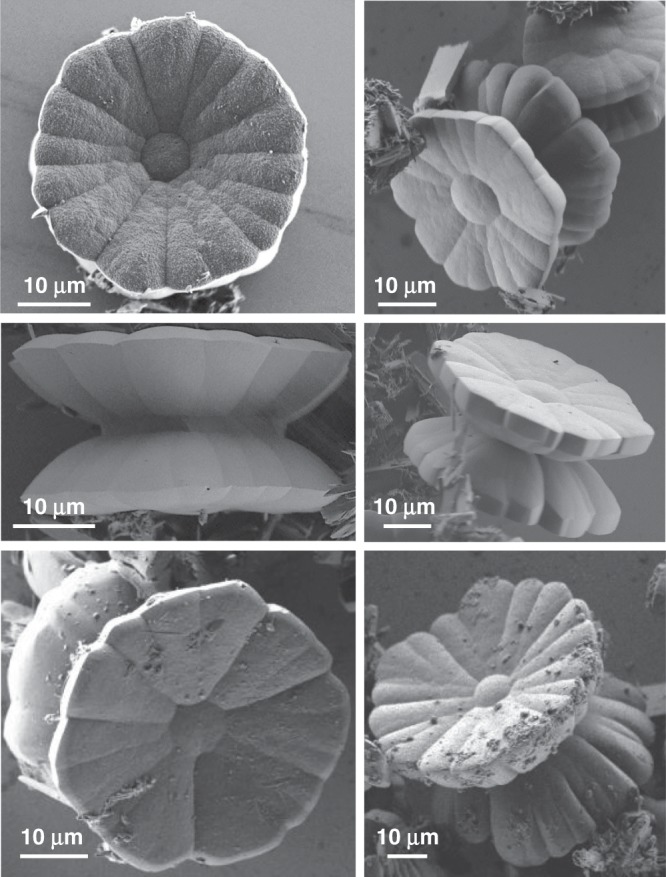
Yo-yo-like single crystals. Scanning electron microscopy (SEM) images recorded after the solvothermal reaction followed by 2 days of aging of the sample at room temperature.

The solvothermal treatment of the achiral organic ligand (TPEPA) at 105 °C, with Cu(NO_3_)_2_·3H_2_O in a 1:2 molar ratio in a chloroform/dimethylformamide (DMF) solution (1:3 v/v) for two days, was followed by two days of aging at room temperature. This procedure resulted in the formation of a green precipitate (Fig. 1a). SEM images revealed the formation of structures having a unique yo-yo-like morphology consisting of an axle and two concave disks (Fig. 2). The texture of the bases resembles a flower with well-defined petals and a stigma at the center. The disks have a diameter of 42 ± 8 μm and the corolla consists of 14 ± 3 petals, as indicated by analyzing more than 50 structures. SEM images of the side-view clearly show the presence of petals down to the center of the yo-yo. A small off-set at this center between the corollas of the two disks indicates a chiral morphology (Figs. 1b and 2). In addition, organic crystals of TPEPA are observed as well (vide infra).

**Fig. 3:**
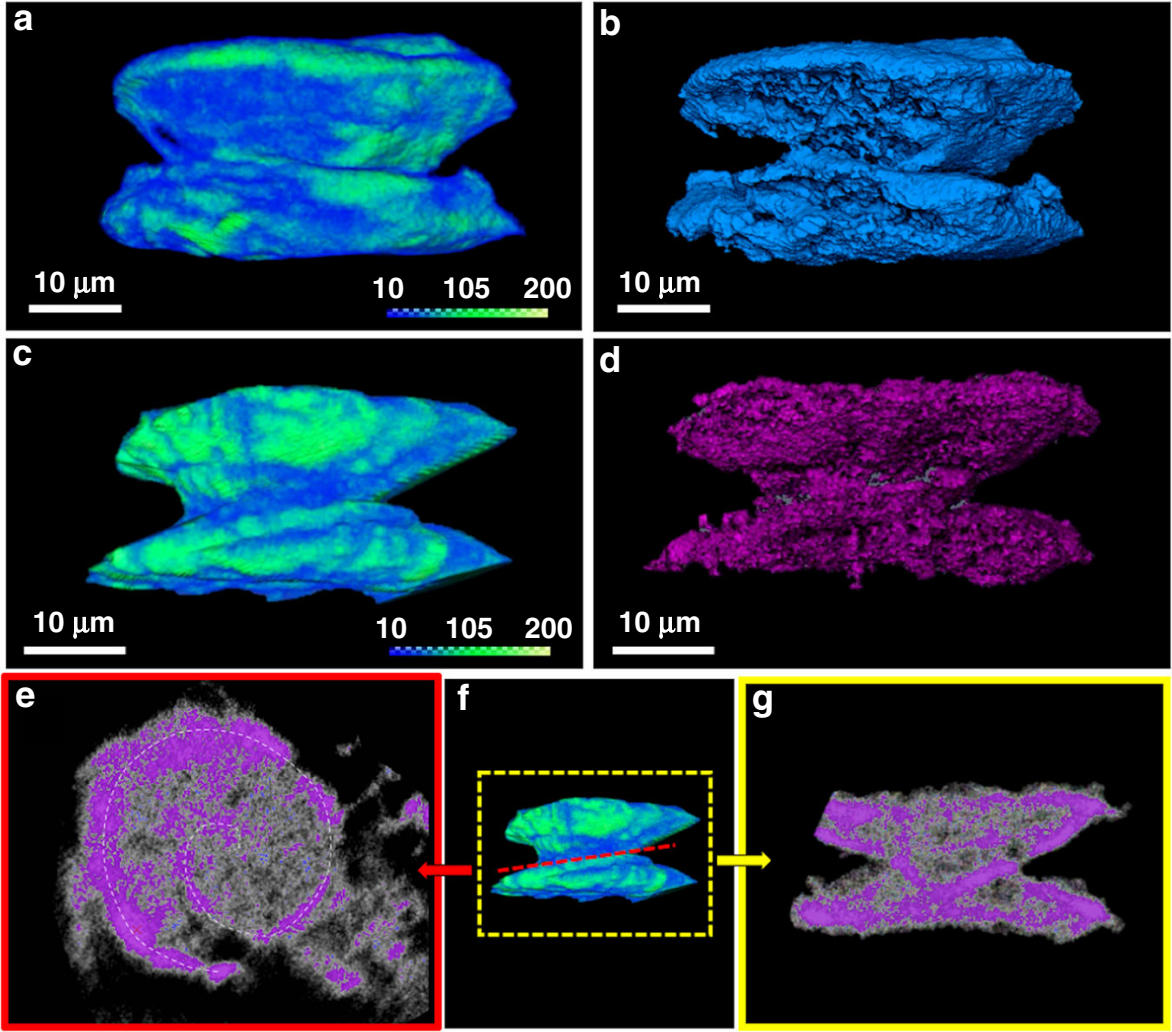
Chirality at the morphological level. Micro-computed tomography measurements showing the off-set angle between the constituting disks of the yo-yo-like crystals. **a**, **c** Volume rendering of two representative crystals after 2 days of aging at room temperature. The color legend shows the Hounsfield units values, which are proportional to the amount of material. **b**, **d** Volume rendering after performing segmentation of the crystals shown in images (**a**), (**c**), respectively. The structure analysis allows to visualize only the higher intensity regions. **e**–**g** Density projections of the sections crossing parallelly (red frame) and perpendicularly (yellow frame) the central region of a crystal. The purple color in (**e**), (**g**) highlights the regions with the highest intensity.

3D volume reconstruction by micro-computed tomography (micro-CT) reveals details about the chiral morphology and growth mechanism, which are not readily observed by other methods (Fig. 3, Supplementary Movies 1 and 2). Different electron-density regions resembling a spiral arrangement are apparent in side-views of these micro-scale objects. The electron-density is relatively low at the core region. The micro-CT data show the opposite chirality of the micro-scale yo-yo-like crystals. To further emphasize these features, we provide additional high-resolution MicroCT images in the Supporting Information that strengthen the chirality aspect at the morphological level (Supplementary Fig. 1). These images also show clearly the spiral motifs.

Cross-section analysis of the x- and y-planes unambiguously shows that the inner arrangement also consists of a continuous single spiral structure. These electron-density regions span the entire structure from base-to-base, as expected for the formation of a single chiral entity. These observations are consistent with spontaneous and chiral morphological growth resulting in crystals having opposite handiness from the two achiral components (TPEPA and Cu(NO_3_)_2_·3H_2_O).

### Single crystallinity and enantiopurity

**Fig. 4:**
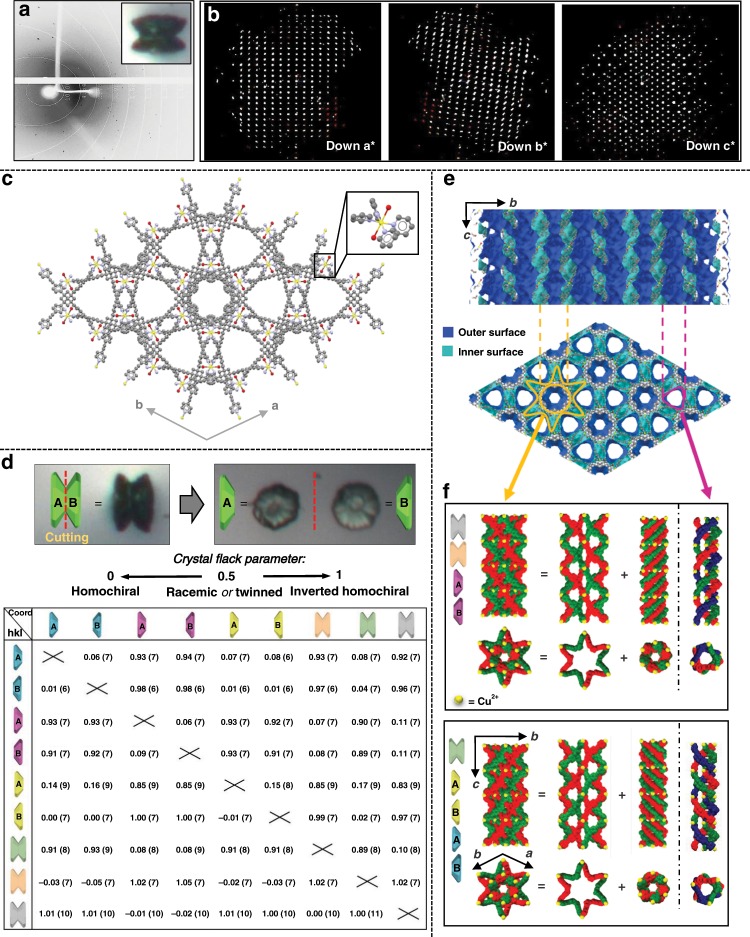
Single crystallinity and chirality. The single crystallinity is definitely assessed by analysis of the X-ray patterns (consisting of well-defined and separated spots): the reflections of the entire yo-yo-like crystal can be indexed with a single domain. The experimental Flack parameters prove that each crystal is enantiopure. The Flack parameters calculated by crossing the hkl data and coordinates of two different crystals (or part of it) show that the bulk is racemic. **a** X-ray experimental diffraction 1 degree frame related to an entire yo-yo-like crystal after 2 days of aging. An optical microscopy image of the analyzed structure is shown in the inset. **b** Projections of the Ewald’s sphere down the a*, b*, and c* axis related to an entire yo-yo-like crystal after 2 days of aging. **c** Ball and stick representation of the crystallographic structure down the c axis; zoom in of the coordination center is reported in the frame on the top right. Color code: C, gray; O, red; N, violet; and Cu, yellow. (**d**, top) Optical microscopy images of the constituting disks (right) obtained after splitting a yo-yo-like crystals after 2 days of aging (left). Schematic representation of each subunit as A or B disk. (**d**, bottom) Flack parameters calculated by crossing the hkl data and coordinates of three entire crystals and three crystals mechanically cut in their constituting disks. **e** Connolly surface representation of the crystal structure down the (top) *a* and (bottom) *c* axes. The channels with triangular and hexagonal geometry are outlined by pink and yellow lines, respectively. **f** Channel helicoids of the crystals whose absolute chirality is analyzed in (**d**). The helicates constituting the channels are reported in blue-red-green and red-green for the triangular and hexagonal channels, respectively.

Our X-ray diffraction studies unambiguously show that the yo-yo-like structures formed after two days of aging are a racemic mixture of single crystals. The surprising single crystallinity of these morphologically highly complex crystals is clearly indicated by the diffraction patterns of several entire structures and of mechanically cut crystal domains (e.g., petals) (Fig. 4). Entire crystals were exposed to a 100 μm X-ray beam. The resulting patterns consist of well-defined and separated diffraction spots, as shown for individual frames. The reflections could be indexed with a single domain without any indication of multiplicity or twinning. The crystal structures were solved and refined to an atomic resolution of 1.05 Å. The space group of these crystals, *P*622, indicated that the molecular packing is chiral. This space group is one of the 65 Sohncke groups and is very rare, with only seven entries^28–34^ currently found in the Cambridge Crystallographic database. Unlike space groups with enantiomers such as *P*6_1_ and *P*6_5_, the enantiomer of *P*622 is also *P*622 making the determination of the absolute chirality of the crystals a challenge. The method we used relies upon a cross-validation using calculated Flack parameters (Fig. 4d).

To verify the unusual single crystallinity (that is reflected also in the enantiopurity), we report both the calculated and experimental Flack parameters of entire yo-yo structures and of the half-structures related to the same yo-yo-like crystal (Supplementary Note 1). The relative chirality of the individual yo-yo-like crystals was determined by X-ray structural analyses of a series of crystals using two different approaches: (I) For three intact yo-yo-crystals, their enantiopurity and single crystallinity were determined by full X-ray structure data collection, solution and refinement. (II) Three more crystals were mechanically cut at the narrow center region into their corresponding halves. All structures (I, II) were completed and refined with its correct handedness (as indicated by a Flack parameter close to 0). To determine the relative handedness of the crystals, the correlating hkl data of one half was refined against the coordinates of the other complementary half, and vice versa: when the handedness is the same the Flack parameter is close to 0, for opposite handedness the Flack parameter is close to 1 (Fig. 4d and Supplementary Tables 2–6). This method allowed us to also determine the chiral composition of the bulk and the absolute configuration of the individual crystals. Correlating hkl data and coordinates of different crystals resulted in Flack parameters approaching zero or one (=opposite chirality). The examined series revealed a racemic mixture of enantiopure crystals.

The chirality originates from the presence of a continuous network of helicates formed by metal-ligand interactions. The Cu^2+^ centers are coordinately saturated and have four pyridine moieties of four TPEPA ligands positioned in the equatorial positions and two water molecules bound with their oxygen atoms in the axial positions. The origin of the chirality is induced by the arrangement of the four pyridine moieties around the metal center that generates a propeller-type conformation^35^. Each unit cells contains propellers having all the same handiness. Crystals having the same handiness have the same propeller-type conformations (Supplementary Fig. 2, Supplementary Movies 3 and 4). For the crystal data shown in Fig. 4c, the metal-oxygen distances are significantly larger (0.7 Å) with respect to the distances expected for a regular octahedron (*d*_axial_ = 2.855 Å and *d*_equatorial_ = 2.000 Å and 2.019 Å). Such a distortion is due to the Jahn-Teller effect and it is characteristic of d^9^ hexacoordinate Cu^2+^.

The yo-yo-like crystals do not show a regular faceted crystal morphology. The relative orientation of the crystal-unit cell axis versus the crystal morphology has been determined. The optical images of the crystal mounted on a MiTeGen loop and the relative orientation of the measured unit cell have been included in the Supporting Information (Supplementary Fig. 3).

The network of helicates in symmetry *P*622 gives rise to hexagonal channels surrounded by six triangular channels. The channels are perpendicular to the crystal surfaces resembling a flower i.e., parallel to the *c* axis. The inner walls that define the differently shaped channels have the same handiness within a single crystal. The hexagonal channels consist of two concentric and intertwined helices with opposite handiness, as can be observed along the *a*-axis (side view). By viewing these outer and inner helices along the *c*-axis (top view): (i) a plane of six copper atoms with TPEPA resembling a star (diameter = 34.943 Å), and (ii) a hexagonal plane of six copper atoms with TPEPA (hexagon diameter = 17.337 Å) are observed, respectively (Figs. 4e, f, Supplementary Movies 5–8). This latter, inner helicate defines the structure and chirality of the hexagonal channel. In the triangular channel, the copper ions are arranged in a plane at the tips of two virtually overlaid equilateral triangles as is the Star of David. For all helices in both types of channels, the pyridine moieties of the TPEPA ligands coordinate to the copper ions on consecutive planes. The diameters of the hexagonal and triangular channels are 8.85 Å and 11.84 Å, respectively. Pores at half-height of the unit cell connect the hexagonal and triangular channels. These structural features result in an overall porosity of 39.7% of the unit cell (the contact surface is calculated by using a spherical probe having a radius of 1.2 Å).

### Development of morphology complexity

**Fig. 5:**
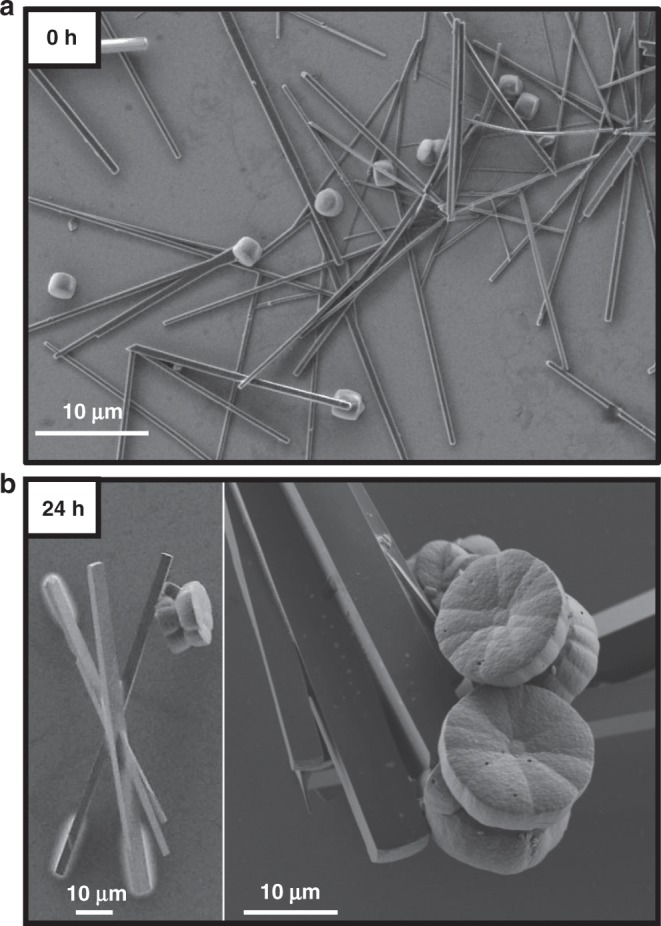
Ligand-based (TPEPA) crystalline rods and yo-yo-like crystals. Scanning electron microscopy (SEM) images of the reaction products recorded **a** after the solvothermal reaction and **b** after 1 day of aging.

**Fig. 6:**
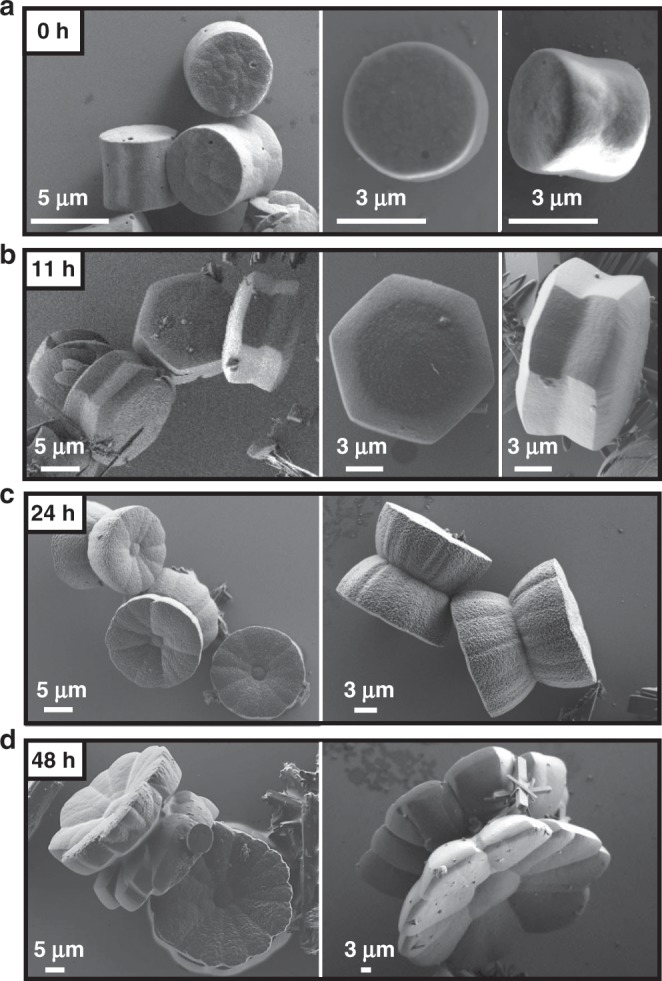
Evolution of the yo-yo-like single crystals as function of the aging time. Scanning electron microscopy (SEM) images. **a** after the solvothermal reaction (0 h), and after **b** 11 h, **c** 1 day and **d** 2 days of room temperature aging.

**Fig. 7:**
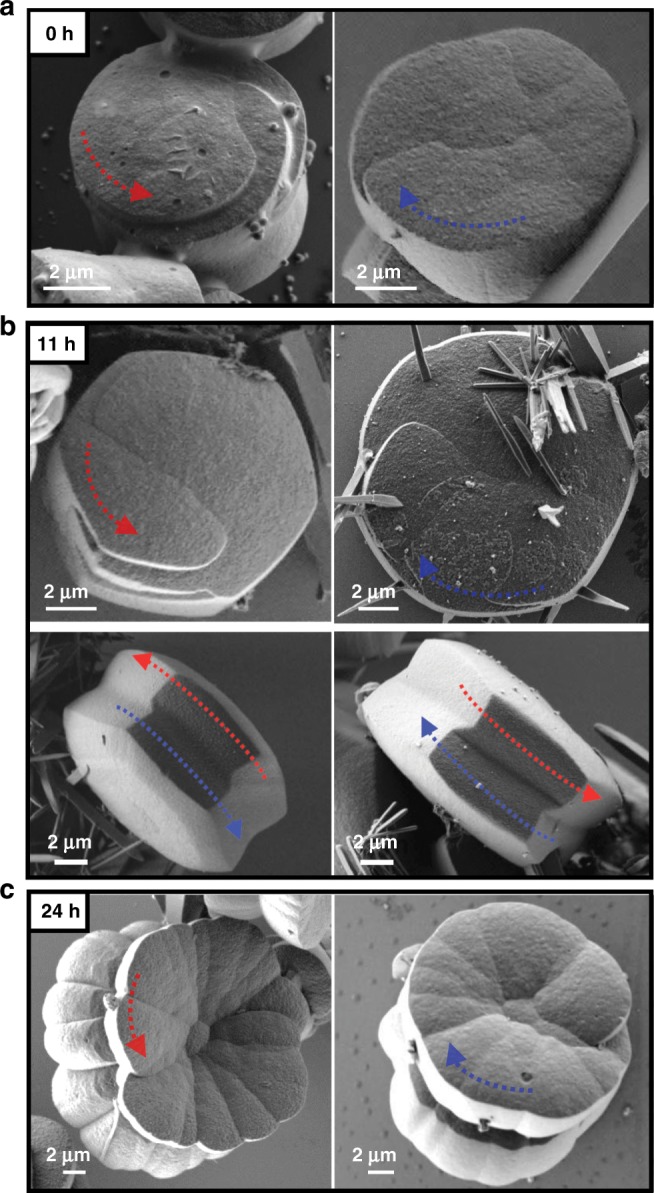
The emergence of chirality at the morphological level. Scanning electron microscopy (SEM) images of metallo-organic structures recorded **a** after the solvothermal reaction and after **b** 11 h, **c** 1 day of aging. The arrows highlight the chirality.

A series of ex situ follow-up scanning electron microscopy (SEM) measurements during aging revealed four distinct stages of the crystallization process, including the step-wise development of the chiral morphology (Figs. 5–7). Stage I: Immediately after the sovothermal reaction, the product solution contained uniform rod-shaped crystals of TPEPA (*l* = 35.6 ± 11.7 μm and ⌀ = 1.17 ± 0.46 μm), fused rods, and cylindrical structures (⌀ = 3.3 ± 1.1 μm and *h* = 2.8 ± 0.8 μm) (Fig. 5a and Supplementary Fig. 4). These structures undergo a series of morphological transformations and dimensional changes in the reaction solution during several days at room temperature. After one day of aging, SEM analysis revealed that the rods form elongated structures that reach up to *l* ≈ 200 μm and ⌀ ≈ 30 μm (Fig. 5b). Raman measurements, Transmission Electron Microscopy (TEM) elemental mapping and structural refinement from X-ray diffraction data (of a two-day aged rod) show that these structures are organic crystals of TPEPA (Supplementary Table 1, Supplementary Figs. 5–7). In parallel to the development of the rods, we observed the crystal growth and evolution of the metal-containing yo-yo-like structures at room temperature from uniform cylinders obtained under solvothermal conditions. These cylindrical structures make up ~35% of the assemblies found in the samples. Both unbound cylindrical structures and structures attached to the rods are present (Fig. 5, Supplementary Figs. 4a, d, f). We observed that some of the cylindrical structures are narrower at the center in the height direction (Fig. 6a). Stage II: After 11 h of aging, the cylindrical structures are converted into prismatic objects having a hexagonal base (Fig. 6b and Supplementary Fig. 8). This morphological change is accompanied by a concurrent increase in dimensions (⌀ = 11.3 ± 2.0 μm and *h* = 4.9 ± 0.8 μm). The hexagonal profile at the microscale is a manifestation of the hexagonal geometry of the unit cell (*P*622) at the molecular level (vide supra). Interestingly, two enantiomeric structures emerge, resembling hexagons connected with their base having an off-set angle (Fig. 6b, right; Fig. 7b, bottom; Supplementary Fig. 8). We succeeded to capture a few structures having loops present at their hexagonal base (Fig. 7b top). These features are consistent with spiral growth around a screw dislocation^36–39^ and are in excellent agreement with the microCT measurements (Fig. 3). Stage III**:** After 24 h we observed further crystal growth and striking morphological changes, including the chiral growth of petals, both in a clock and anticlockwise manner (Figs. 6c, 7c and Supplementary Fig. 9). At this stage, the development of texture implies the start of a new, parallel growth process. This effect might be caused by lowering the concentrations of TPEPA and the copper salt during the aging process^8,40^. However, continuous growth is still observed for an additional 24 h, meaning that the reaction solution is still within the supersaturation range. In addition, birth and spread growth was also observed (Supplementary Fig. 10). The EDS data of these flower structures show the homogeneous distribution of nitrogen and copper, as expected for the formation of ordered metallo-organic assemblies (Supplementary Fig. 11, Supplementary Note 2). Stage IV: After 48 h, the formed crystals are stable and the chiral arrangement of the petals is not apparent (Fig. 6d and Supplementary Fig. 12). Prolonged reaction times do not alter the appearance of the crystals.

Ex situ Raman measurements indicate that during these four stages of crystal growth the nitrogen atoms of the pyridine moieties are coordinated to the copper cations (Supplementary Fig. 5). The coordination is indicated by a change in the relative intensity ratio of two characteristic bands of TPEPA at *ν* = 1593 and 1607 cm^−1^ (pyridine modes). Moreover, the initially formed cylindrical structures during Stage I have relatively broad Raman peaks at *ν* = 1150 (benzene ring mode) and 1607 cm^−1^ (pyridine mode). The width of these two signals decreases in time and the intensity of the peaks at *ν* = 1150 cm^−1^ and *ν* = 2225 cm^−1^ (C≡C stretching) increases by ~30% and ~67%, respectively. These observations suggest an increase in the crystallinity during the aging process.

## Discussion

We provided here a comprehensive and detailed mechanistic and structural analysis of the growth of multidomain crystals possessing chirality at different levels (morphology and packing). An unexpected finding of our study is the extreme complexity of the unusual crystal appearance combined with single crystallinity. This combination in one crystal is very unusual. The individual crystals are enantiopure and their homochiral channels are continuous as indicated by the single crystallinity of our crystals. The origin of the chiral packing (of the achiral components) is a result of the propeller-type arrangement of the pyridine units coordinated to the copper ion. A direct connection between the chirality of helicates and the morphology is not obvious, the chiral morphology evolved from apparent achiral cylindrical structures. The final yo-yo-like structure consist of two constituting disks that resemble flowers with petals. The asymetric morphology evolves through spiral growth processes. To this end, these crystals can be considered a new kind of chiral and porous materials. The rich coordination chemistry of pyridine-based ligands with metal salts offers ample opportunities to generate a new class of fascinating multidomain, single crystals.

## Methods

### Materials

Chloroform (CHCl_3_, ≥99.8%) and dimethylformamide (DMF, ≥99.8%) were purchased from Sigma Aldrich and J. T. Baker, respectively. Cu(NO_3_)_2_∙3H_2_O (>98.0%) was purchased from Fluka. Reagents were used without further purification. TPEPA was synthesized according to a literature procedure^41^. Glass pressure tubes (Ace Glass Inc., pressure tubes #15 with plunger valve, PTFE Bushing and FETFE^®^ O-Ring, volume 50 mL) were cleaned by immersion in a H_2_SO_4_/30% H_2_O_2_ piranha solution (7:3 v/v) for 10 min. Subsequently, they were washed with deionized (DI) water and dried in an oven for 12 h at 130 °C. Caution: piranha is an extremely dangerous oxidizing agent and should be handled with care using appropriate personal protection. Elemental analyses (C, H, N, Cl, and Cu) were performed at Kolbe Laboratorium, Mulheim, Germany. The oxygen is calculated as the leak to 100%. The reported values are the average of duplicate measurements. The error is ±0.01% for C,H,N; ±0.02% for Cl and Cu. The infrared spectra were obtained using a Nicolet 460 single beam Fourier transform infrared spectrophotometer (FT-IR).

### Preparation of yo-yo-like crystals and TPEPA rod-like crystals

A suspension of TPEPA in CHCl_3_ (3.55 mM, 2.0 mL) was prepared in a 20 mL glass vial. Subsequently, 3.0 mL of DMF was added and the mixture was sonicated for 1.5 h. Cu(NO_3_)_2_∙3H_2_O was dissolved in DMF (7.1 mM; 10 mg in 5.8 mL of DMF). The reaction solution was prepared by transferring 1.0 mL of the Cu(NO_3_)_2_∙3H_2_O containing DMF solution and 3.0 mL of the TPEPA containing CHCl_3_/DMF solution to a glass pressure tube (the final concentrations of TPEPA and Cu(NO_3_)_2_∙3H_2_O are 0.9 mM and 1.8 mM, respectively). The tube was sealed and heated for 48 h at 105 °C without stirring and with the exclusion of light. Then it was allowed to attain room temperature by decreasing the temperature of the oven by using a controller (Lae Electronic, two-channel universal controller, AC1-5). The temperature of the controller was decreased every hour by 10 °C. This process resulted in a light yellow/green precipitate and a yellowish solution. The system was left to age at room temperature in the mother liquor for 2 days. During the aging process the green color of the precipitate increased.

### Scanning electron microscopy (SEM)

SEM measurements were performed using HRSEM ULTRA-55 ZEISS and HRSEM SUPRA-55 VP ZEISS instruments at an EHT voltage of 1.5/3 kV. Images were collected in secondary electron modes by using Everhart-Thornley detector. SEM samples were prepared by placing a drop of the reaction mixture on a silicon substrate and drying under air.

### Micro-computed tomography (MicroCT)

MicroCT measurements were performed by using a Micro-XCT400 Zeiss (Fig. 3) and Xradia 520 Versa Zeiss (Fig. S1) X-ray microscopes (Peasanton, California, USA). The samples were dried overnight under vacuum. A plastic pipette tip was used as sample container: the narrowest extremity of the pipette tip was melted and sealed using a flame. Subsequently, the dried sample was added to the tip. The tomographic images performed by the Micro-XCT400 Zeiss microscope were obtained by collecting 1200 projections over 180 deg at 40 KV and 200 µA. The final pixel size was 0.33 µm. The tomographic images performed by the Xradia 520 Versa Zeiss microscope were obtained by taking 1601 projections over 360 deg at 45 KV and 67 µA. The final pixel size was 0.613 µm. Collective 3D images of the samples were obtained. Subsequently, detailed analysis of several individual structures were performed by using the Avizo 9.5 software (Thermo Fisher Scientific Inc, USA).

### Raman spectroscopy

Raman measurements were conducted on a LabRAM HR Evolution instrument (Horiba, France). The instrument is equipped with an 800 mm spectrograph and a CCD detector (1024 × 256 pixels open electrode front illuminated CCD camera, cooled to −60 °C). The system is set around an open confocal microscope (BX-FM Olympus, Japan). The measurements were performed using a 632.8 nm HeNe laser, with 600 grooves/mm grating and a ×100 objective (spatial resolution better than 1 μm). The pixel spacing is 1.3 cm^−1^.

### Transmission electron microscopy (TEM)

Cross-sectional samples for TEM investigation were prepared using focused ion beam milling with Ga^+^ ions in a FEI Helios FIB instrument and by microtome sectioning from samples embedded in resin (Epon). TEM images and diffraction data were recorded in a FEI Tecnai T12 instrument operated at 120 kV. STEM images and EDS maps were obtained at an accelerating voltage of 80 kV in a probe aberration-corrected FEI Titan G2 60–200 ChemiSTEM microscope^42^ equipped with a high-brightness FEG. EDS spectrum image data were obtained with a Bruker Super-X four-segment SDD detector with a probe current of 120 pA and a total recording time of about 300 s.

### Single-crystal X-ray diffraction (SXRD)

SXRDs were collected both by synchrotron source at the Beamline ID-29 of European Synchrotron Radiation Facility (ESRF) and by a Rigaku XtaLabPro X-ray diffractometer. The Rigaku XtaLabPro X-ray diffractometer is equipped with a 4-circle Kappa goniometer, a Dectris Pilatus 3R 200K-A detector, and a micro-focus sealed tube with microCMF-VHF. The data were collected with *λ* = 0.700 Å (syncrhroton) and CuKα1 radiation, *λ* = 1.5418 Å (Rigaku XtaLabPro diffractometer). The Rigaku XtaLabPro instrument presents a micro-focus beam with a 100 μm beam; such a beam is large enough to expose the entire crystal. All the crystals were placed in Hampton Paratone oil, mounted on a MiTeGen loop and plunged into LN to flash freeze. The data were collected at 100 K with Oxford Cryostream. The crystals analyzed at the synchrotron were transported frozen in a Taylor-Wharton CX100 dry shipper. Data collection and reduction for the synchrotron data were done using MXCuBE^43^, and the EDNA automated data processing pipeline with XDS. Data collection, reduction and analysis for the XtaLabPro laboratory data were performed with the CrysAlisPro software package (version 1.171.39.22a). The crystal structures were solved by direct methods using SHELXT 2016/4^44^. All non-hydrogen atoms were further refined by SHELXL with anisotropic displacement coefficients^44^. Hydrogen atoms were assigned isotropic displacement coefficients, and their coordinates were allowed to ride on the respective carbon atoms. Platon SQUEEZE protocol was applied for all the structures. Mercury CSD 3.10.2 and PLATON software were used for graphics. Several types of crystals were analyzed: a rod-like crystal, entire yo-yo-like crystals and parts of them, i.e., a single “petal” and half yo-yo-like crystals (constituting disks). Rod-like crystal: A colorless rod-like crystal was taken from the sample containing also the 2 days aged yo-yo-like crystals. The rod-like crystal was coated in Hampton Paratone oil, mounted on a MiTeGen loop and flash frozen in the Oxford Cryosystem stream. The crystal was measured by the Rigaku XtaLabPro diffractometer. Data were collected using CrysAlisPro 1.171.39.46. Data were collected at 100 K as 0.5° ω scans with a 100 μm beam. The structure solution and refinement were carried out using the SHELXT algorithms in Olex2^45^ and with SHELXL-2016/4^44^. Crystal data and details of the structure refinement revealed that it comprises only the organic ligand. For details, see Supplementary Table 1, CCDC 1949924. Entire yo-yo-like crystals: three entire yo-yo-like crystals were measured by the Rigaku XtaLabPro X-ray diffractometer. The X-ray diffraction data and CrysAlisPro software showed no indication of twinning (i.e., unit cell was readily found and a high percentage of all reflections >90% could be readily indexed in that unit cell without any twin domain being found). The structures were solved and fully refined to reasonable resolution and R factors by using SHELXL 2016/4^44^. For details, see Supplementary Table 2 and CCDCs 1910232, 1910233, and 1910231. Half yo-yo-like crystals (constituting disks): the half yo-yo-like crystals were measured both by the Rigaku XtaLabPro diffractometer and synchrotron source. In order to obtain the two half structures, the entire MOFs were placed in Hampton Paratone oil and cleaved down the center (where the structure is most narrow) under the microscope by using a fine needle, MiTeGen micro tools and loops. Subsequently each half was mounted on a MiTeGen loop and flash frozen as previously reported. Three different full double-flowers were cleaved and collected (a total of six half flower data sets) by the Rigaku XtaLabPro X-ray diffractometer. A further half one was analyzed at the synchrotron for crystal structure confirmation. The diffraction data showed no indication of twinning (i.e., unit cell was readily found and a high percentage of all reflections >90% could be readily indexed in the that unit cell without any twin domain being found). The structures were solved and fully refined to reasonable resolution and R factors by using SHELXL 2016/4^44^. For details, see Supplementary Tables 3–5, CCDCs 1910239, 1910238, 1910234, 1910237, 1910236, and 1910235. Petal region: a yo-yo-like crystal was placed in Hampton Paratone oil and a small piece of a single wedge-shaped “petal” was cut off under the microscope, by using a fine needle and MiTeGen micro tools. The piece was mounted on a MiTeGen loop and flash frozen as previously reported. Data were collected at ESRF ID29 as 270 frames of 1.0° ϕ scans with a 30 μm beam. The structure solution and refinement were carried out by using the SHELX algorithms in Olex2 and with SHELXT-2016/4^44^. (For details, see Supplementary Table 6, CCDC 1910230).

## Supplementary information

Supplementary Information

Description of Additional Supplementary Files

Supplementary Movie 1

Supplementary Movie 2

Supplementary Movie 3

Supplementary Movie 4

Supplementary Movie 5

Supplementary Movie 6

Supplementary Movie 7

Supplementary Movie 8

## Data Availability

The authors declare that the data supporting the findings of this study are available within the paper and its supplementary information files. The crystallographic data has also been submitted in the database Cambridge Crystallographic Data Centre. The CCDC numbers are 1949924, 1910230-1910239.
