# Supplementary material for: Emergence of chirality and structural complexity in single crystals at the molecular and morphological levels
Source: Nat Commun. 2020 Jan 20;11:380. doi: 10.1038/s41467-019-13925-5 (PMC6971082; doi:10.1038/s41467-019-13925-5)
Supplement: Supplementary file 1 — Supplementary Information [file 41467_2019_13925_MOESM1_ESM.pdf]

Supplementary Informations

Emergence of chirality and structural complexity in single crystals at the molecular and morphological levels

di Gregorio et al.

Compound	Rod-like crystal
CIF file name	V344
CCDC	1949924
Crystal description	Yellow needle
Diffractometer	Rigaku XtaLabPro
Empirical formula	$C_{62}H_{44}N_4$ + [solvent]
Formula weight (g/mol)	845.01
Temperature (K)	100
Wavelength (Å)	1.54184
Crystal system	*Tetragonal*
Space group	$I4_1/a$
a (Å)	26.7291(4)
b (Å)	26.7291(4)
c (Å)	7.05940(15)
α,β, γ°	90, 90, 90
Volume (Å^3)	5043.6(2)
Z	4
Density (Mg/m^3)	1.113
Absorption coefficient (mm^{-1})	0.499
F(000)	1776.0
θ range for data collection (°)	2.34-28°
Reflection collected (Unique)	5645 (1649)
R int	0.0302
Completeness %	99.8
Data\restraints\ parameters	1649/0/150
Goodness-of-fit on F^2	1.10
Final R [I>2σ(I)]	R_1=0.0565 wR_2=0.1779
R (all data)	R1=0.0632 wR2=0.1861
Largest diff. peak and hole (e$^-$ Å^{-3})	0.137 and 0.200

Supplementary Table 1. Single crystal X-ray data for a rod-like crystal. Crystal data and structure refinement parameters for a rod-like crystal forming after two days of solvothermal reaction followed by 2 days of aging at room temperature.

Crystal and symbol	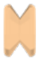	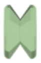	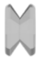
CIF file name	V295	V297	V305
CCDC	1910232	1910233	1910231
Crystal description	Green yo-yo	Green yo-yo	Green yo-yo
Diffractometer	Rigaku XtaLabPro	Rigaku XtaLabPro	Rigaku XtaLabPro
Empirical formula*	$C_{62}H_{48}CuN_4O_2$ + 2[NO_3] +[SOLVENT]	$C_{62}H_{48}CuN_4O_2$ + 2[NO_3] +[SOLVENT]	$C_{62}H_{48}CuN_4O_2$ + 2[NO_3] +[SOLVENT]
Formula weight (g/mol)	1068.60	1068.60	1068.60
Temperature (K)	100	100	100
Wavelength (Å)	1.54184	1.54184	1.54184
Crystal system	Hexagonal	Hexagonal	Hexagonal
Space group	*P622*	*P622*	*P622*
a (Å)	26.255(1)	26.573(2)	26.158(4)
b (Å)	26.255(1)	26.573(2)	26.158(4)
c (Å)	18.174(1)	17.807(1)	18.105(3)
α, β, γ°	90,90,120	90,90,120	90,90,120
Volume ($Å^3$)	10849.5(2)	10889.3(17)	10729(4)
Guest accessible volume (% unit cell)**	40.4	41.1	40.2
Z	6	6	6
Density (Mg/m^3)	0.981	0.978	0.992
Absorption coefficient (mm^{-1})	0.804	0.801	0.813
F(000)	3330.0	3330.0	3330.0
θ range for data collection (°)	4.154 to 47.866	4.152 to 47.778	4.170 to 44.491
Reflection collected (Unique)	13835 (3379)	10526 (3359)	9112 (2831)
R int	0.0756	0.0574	0.0702
Completeness %	99.2	98.6	99.4
Data\restraints\parameters	3379/0/283	3359/16/268	2831/128/259
Goodness-of-fit on F^2	0.905	0.963	0.976
Final R [I>2σ(I)]	R_1=0.0842 wR_2=0.2216	R_1=0.0895 wR_2=0.2460	R_1=0.0961 wR_2=0.2644
R (all data)	R_1=0.1275, wR_2=0.2509	R_1=0.1220 wR_2=0.2682	R_1=0.1349 wR_2=0.2927
Largest diff. peak and hole ($e^- Å^{-3}$)	0.261 and -0.258	0.467 and -0.388	0.244 and -0.226
Exp. Flack Parameter	0.09(8)	-0.02(7)	0.00(11)

* The nitrate counter ions have not been observed by X-ray analysis, but have been included in the formula for completeness. These ions have been observed spectroscopically by FT-IR (Supplementary Fig. 13)
** Calculated using the "contact surface", obtained from the Mercury CSD 3.10.2 program, employing a spherical probe of 1.2 Å radius.

Supplementary Table 2. Single crystal X-ray data for entire yo-yo-like crystals. Crystal data and structure refinement parameters for the 3 intact *yo-yo*-like crystals indicated in Figure 4 with orange, green and grey symbols, respectively.

Crystal and symbol	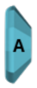	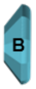
CIF file name	V286	V287
CCDC	1910239	1910238
Crystal description	Green half yo-yo	Green half yo-yo
Diffractometer	Rigaku XtaLabPro	Rigaku XtaLabPro
Empirical formula*	$C_{62}H_{48}CuN_4O_2$ + 2[NO_3] +[SOLVENT]	$C_{62}H_{48}CuN_4O_2$ + 2[NO_3] +[SOLVENT]
Formula weight (g/mol)	1068.60	1068.60
Temperature (K)	100	100
Wavelength (Å)	1.54184	1.54184
Crystal system	hexagonal	hexagonal
Space group	*P622*	*P622*
a (Å)	26.7410(15)	26.7674(17)
b (Å)	26.7410(15)	26.7674(17)
c (Å)	17.8929(10)	17.8783(15)
α, β, γ°	90,90,120	90,90,120
Volume ($Å^3$)	11080.7(14)	11093.5(17)
Guest accessible volume (% unit cell)**	42.2	41.9
Z	6	6
Density (Mg/m^3)	0.961	0.960
Absorption coefficient (mm^{-1})	0.788	0.787
F(000)	3330.0	3330.0
θ range for data collection (°)	4.128 to 47.778	4.126 to 47.795
Reflection collected (Unique)	14230 (3443)	23394(3431)
R int	0.0550	0.0708
Completeness %	99.7	98.9
Data\restraints\parameters	3443/6/316	3431 \ 90 \ 283
Goodness-of-fit on F^2	1.021	0.978
Final R [I>2σ(I)]	R_1=0.0856 wR_2=0.2320	R_1=0.0870 wR_2=0.2283
R (all data)	R_1=0.1187 wR_2=0.2522	R_1=0. 1201 wR_2=0.2497
Largest diff. peak and hole ($e^- Å^{-3}$)	0.498 and -0.289	0.501 and -0.465
Experimental Flack Parameter	0.07(7)	0.01(6)

* The nitrate counter ions have not been observed by X-ray analysis, but have been included in the formula for completeness. These ions have been observed spectroscopically by FT-IR (Supplementary Fig. 13).
* Calculated using the "contact surface", obtained from the Mercury CSD 3.10.2 program, employing a spherical probe of 1.2 Å radius.

Supplementary Table 3. Single crystal X-ray data for half yo-yo-like crystal. Crystal data and structure refinement parameters for the two subunits of the yo-yo-like crystals indicated in Figure 4 as blue symbols (A and B).

Crystal and symbol	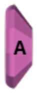	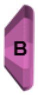
CIF file name	V288	V290
CCDC	1910234	1910237
Crystal description	Green half yo-yo	Green half yo-yo
Diffractometer	Rigaku XtaLabPro	Rigaku XtaLabPro
Empirical formula*	$C_{62}H_{48}CuN_4O_2 + 2[NO_3]$ +[SOLVENT]	$C_{62}H_{48}CuN_4O_2 + 2[NO_3]$ +[SOLVENT]
Formula weight (g/mol)	1401.38	1068.60
Temperature (K)	100	100
Wavelength (Å)	1.54184	1.54184
Crystal system	hexagonal	hexagonal
Space group	*P622*	*P622*
a (Å)	26.619(4)	26.6912(17)
b (Å)	26.619(4)	26.6912(17)
c (Å)	17.897(3)	17.9351(12)
α, β, γ°	90, 90, 120	90, 90, 120
Volume (Å^3)	10982(3)	11065.5(16)
Guest accessible volume (% unit cell)**	41.9	42.4
Z	6	6
Density (Mg/m^3)	1.271	0.962
Absorption coefficient (mm^{-1})	2.770	0.789
F(000)	4212.0	3330.0
θ range for data collection (°)	4.139 to 47.867	4.129 to 47.800
Reflection collected (Unique)	14343(3422)	14548(3424)
R int	0.0515	0.0559
Completeness %	99.4	99.0
Data/restraints/parameters	3422/0/316	3424/90/301
Goodness-of-fit on F^2	0.976	1.024
Final R [I>2σ(I)]	R_1=0.0877 wR_2=0.2337	R_1=0.0836 wR_2=0.2306
R (all data)	R_1=0.1131 wR_2=0.2563	R_1=0.1175 wR_2=0.2518
Largest diff. peak and hole (e$^-$ Å^{-3})	1.230 and -0.335	0.499 and -0.298
Experimental Flack Parameter	0.07(7)	0.07(7)

* The nitrate counter ions have not been observed by X-ray analysis, but have been included in the formula for completeness. These ions have been observed spectroscopically by FT-IR (Supplementary Fig. 13).
* Calculated using the "contact surface", obtained from the Mercury CSD 3.10.2 program, employing a spherical probe of 1.2 Å radius.

Supplementary Table 4. Single crystal X-ray data for half yo-yo-like crystal. Crystal data and structure refinement parameters for the two subunits of the yo-yo-like crystals indicated in Figure 4 as pink symbols (A and B).

Crystal and symbol	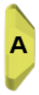	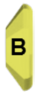
CIF file name	V292	V293
CCDC	1910236	1910235
Crystal description	Green half yo-yo	Green half yo-yo
Diffractometer	Rigaku XtaLabPro	Rigaku XtaLabPro
Empirical formula*	$C_{62}H_{48}CuN_4O_2 + 2[NO_3]$ +[SOLVENT]	$C_{62}H_{48}CuN_4O_2 + 2[NO_3]$ +[SOLVENT]
Formula weight (g/mol)	1068.60	1068.60
Temperature (K)	100	100
Wavelength (Å)	1.54184	1.54184
Crystal system	Hexagonal	hexagonal
Space group	*P622*	*P622*
a (Å)	26.3073(16)	26.6103(19)
b (Å)	26.3073(16))	26.6103(19)
c (Å)	18.1174(12)	17.9240(11)
α, β, γ°	90, 90, 120	90, 90, 120
Volume (Å^3)	10858.7(15)	10991.7(17)
Guest accessible volume (% unit cell)**	41.1	41.1
Z	6	6
Density (Mg/m^3)	0.980	0.969
Absorption coefficient (mm^{-1})	0.804	0.794
F(000)	3330.0	3330.0
θ range for data collection (°)	4.153 to 44.491	4.138 to 47.936
Reflection collected (Unique)	12982 (2873)	12473(3370)
R int	0.0665	0.0503
Completeness %	99.5	98.5
Data/restraints/parameters	2873/6/295	3370/0/289
Goodness-of-fit on F^2	0.987	0.941
Final R [I>2σ(I)]	R_1=0.0802 wR_2=0.2080	R_1=0.0895 wR_2=0.2487
R (all data)	R_1=0.1140 wR_2=0.2327	R_1=0.1215 wR_2=0.2710
Largest diff. peak and hole (e$^-$ Å^{-3})	0.274 and -0.209	0.494 and -0.528
Experimental Flack Parameter	0.16(9)	0.01(7)

* The nitrate counter ions have not been observed by X-ray analysis, but have been included in the formula for completeness. These ions have been observed spectroscopically by FT-IR (Supplementary Fig. 13).

* Calculated using the "contact surface", obtained from the Mercury CSD 3.10.2 program, employing a spherical probe of 1.2 Å radius.

Supplementary Table 5. Single crystal X-ray data for half yo-yo-like crystal. Crystal data and structure refinement parameters for the two subunits of the "*yo-yo*"-like crystals indicated in Figure 4 as yellow symbols (A and B).

Crystal	petal region
CIF file name	V224
CCDC	1910230
Crystal description	Piece of half yo-yo
Diffractometer	ESRF ID29
Empirical formula*	$C_{62}H_{48}CuN_4O_2$ + 2[NO_3] + 0.17[H_2O] + [SOLVENT]
Formula weight (g/mol)	1071.60
Temperature (K)	100
Wavelength (Å)	0.70
Crystal system	Hexagonal
Space group	*P622*
a (Å)	26.140(4)
b (Å)	26.140(4)
c (Å)	18.090(4)
α, β, γ°	90, 90, 120
Volume ($Å^3$)	10705(5)
Guest accessible volume (% unit cell)**	39.7
Z	6
Density (Mg/m^3)	0.997
Absorption coefficient (mm^{-1})	0.340
F(000)	3340.0
θ range for data collection (°)	1.109 to 21.609
Reflection collected (Unique)	66163(4382)
R int	0.0634
Completeness %	99.8
Data/restraints/parameters	4382/0/309
Goodness-of-fit on F^2	1.051
Final R [I>2σ(I)]	R_1=0.0641 wR_2=0.1918
R (all data)	R_1=0.0698, wR_2=0.1987
Largest diff. peak and hole ($e^- Å^{-3}$)	0.319 and -0.213

* The nitrate counter ions have not been observed by X-ray analysis, but have been included in the formula for completeness. These ions have been observed spectroscopically by FT-IR (Supplementary Fig. 13).
** Calculated using the "contact surface", obtained from the Mercury CSD 3.10.2 program, employing a spherical probe of 1.2 Å radius.

Supplementary Table 6. Single crystal X-ray data for a petal of a yo-yo-like crystal.
Crystal data and structure refinement parameters for a petal region of a yo-yo-like crystal.

Supplementary Note 1. Single crystal X-ray data and Flack parameter analysis. Full data sets for each crystal including Friedel pair reflections were collected. The Friedel pairs were not merged. The full least square refinement was carried out until convergence for each structure using SHELXL 2016/4.[1] The absolute structure of each completed structure was determined as being correct for that structure by having a Flack parameter close to 0. The Flack parameters were determined using SHELXL $[(I+)-(I-)]/[(I+)+(I-)]^2$ and were taken from the cif files. Such experimental Flack parameters indicated the enantiopurity of each crystal but not the absolute chiral configurations. The absolute chiral configurations for each crystal vs other crystals were done by doing a Cross-Flack refinement. For each pair of structures to be compared (here named crystals 1 and 2), the reflections (hkl) file of crystal 1 was refined until convergence against the coordinate model (xyz) of crystal 2. The resulting Flack parameter x was calculated in SHELXL 2016/4[2] and taken from the cif file. The files were then reversed and the coordinate file (xyz) of crystal 1 was refined to convergence against the reflections (hkl) file of crystal 2. In cases where the Flack parameter was close to 0, it was determined that the relative absolute configuration of the two crystals was the same. In cases where the Flack parameter was close to 1 it was determined that the two crystals were of opposite absolute configuration. These Cross-Flack refinements were done for all possible combinations of crystal structures presented in this paper and are given in the table in Figure 4D.

Supplementary Note 2. Elemental composition. The sample contains after 2 days of ageing both the yo-yo-like structures and organic rods. The bulk elemental composition (weight %) from duplicate elemental analysis is C, 29.35; H, 5.72; N, 3.12; Cl, 1.18; Cu, 2.89. The amount of oxygen is estimated as 57.74%. This composition indicates a formula of $C_{54.4}H_{127}N_5Cu_1Cl_{0.7}O_{80}$. Quantitative analysis of TEM-EDX data was performed on a microtome sliced lamella of a yo-yo-like crystal (Supplementary Fig. 11). A sliced sample was used to avoid possible artifacts due to surface residues or reabsorption of the signal from the low energy carbon X-rays. These TEM-EDX measurements indicate a molecular formula of $C_{41}N_{2.2}Cu_1O_{5.9}$. Hydrogen atoms cannot be detected by this method. We used a ratio of $Cu(NO_3)_2 \cdot H_2O$:TPEPA = 2:1, whereas the crystallographic studies of the yo-yo-like structures reveal a ratio of 1:1. No copper ions are observed in the crystallographic structure of the organic rods. This data suggest that an excess of copper ions is present in the channels of the samples. FT-IR measurements show a band at 1382 cm^{-1} indicative of the presence of nitrate anions (Supplementary Fig. 13).

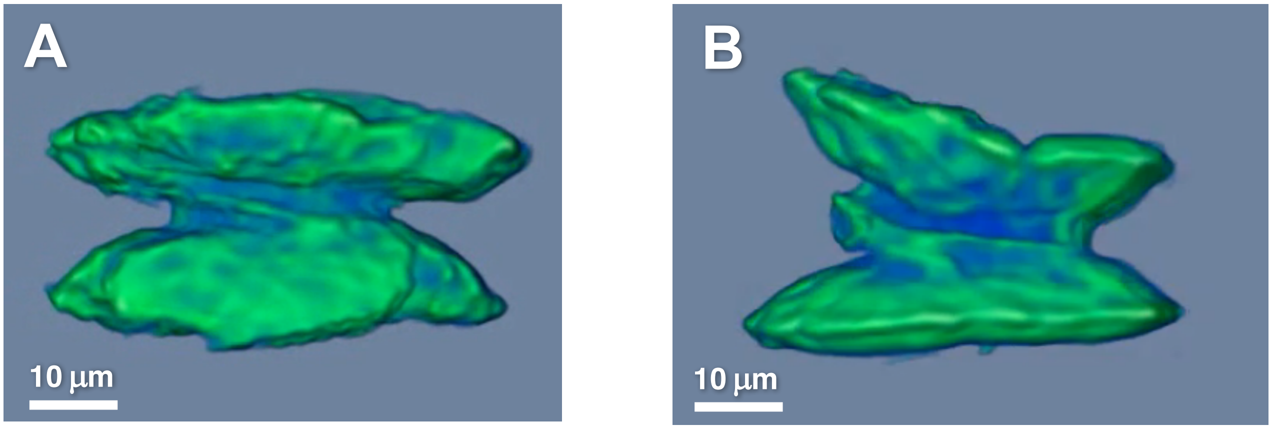

Supplementary Fig. 1. Chirality at the morphological level. Micro-computed tomography measurements showing the off-set angle between the constituting disks of the yo-yo-like crystals. (A, B) Volume rendering of two representative crystals after 2 days of aging at room temperature.

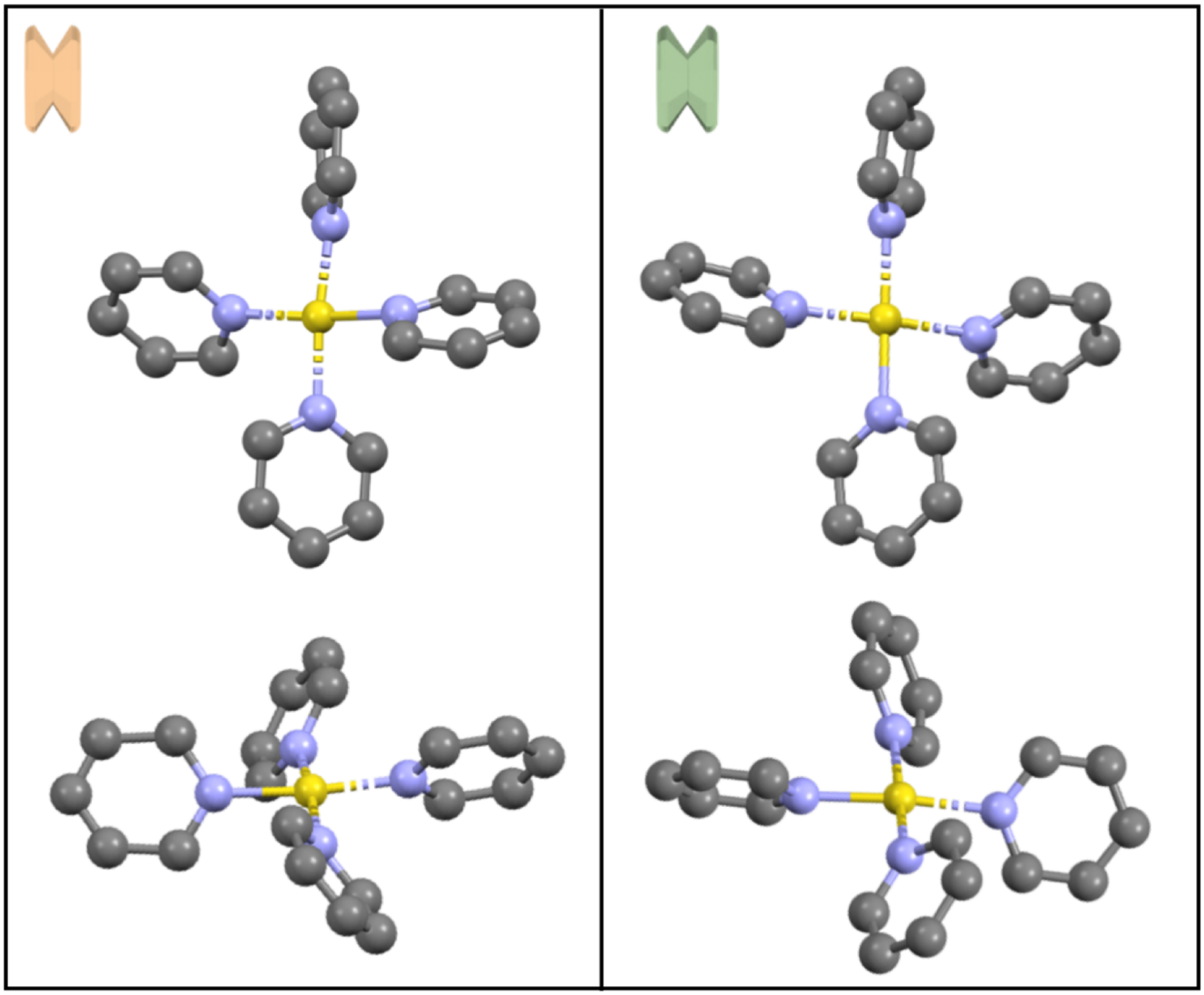

Supplementary Fig. 2. Chirality at the coordination centers. Ball and stick representation of crystallographic structures of two yo-yo-like crystals showing only the arrangement of the four pyridine moieties around the metal center. The left (CCDC1910232) and right images (CCDC1910233) show a propeller geometry with opposite handiness. Color code: C, gray; N, violet; Cu, yellow, H, white.

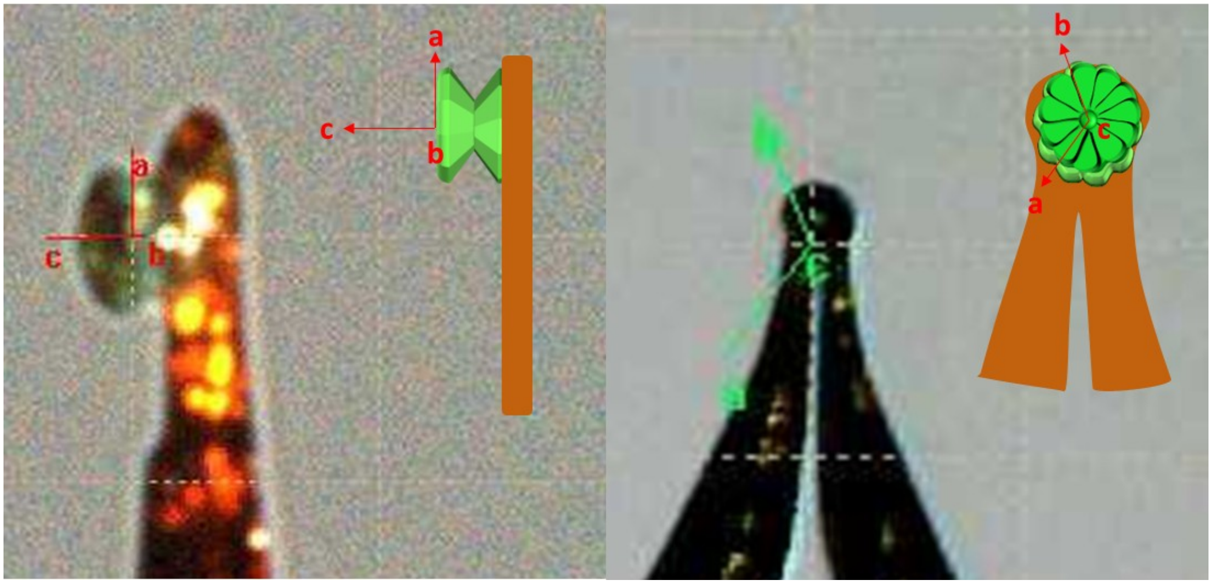

Supplementary Fig. 3. Crystal axes vs crystal morphology. Optical images of yo-yo-like crystals (0.029 mm × 0.049 mm × 0.062 mm) mounted on a MiTeGen loop. The unit cell was determined, and the crystal axes (a, b, c) vs the orientation of the mounted crystal obtained with 'CrysAlisPro 1.171.40.57a (Rigaku OD, 2019)'.

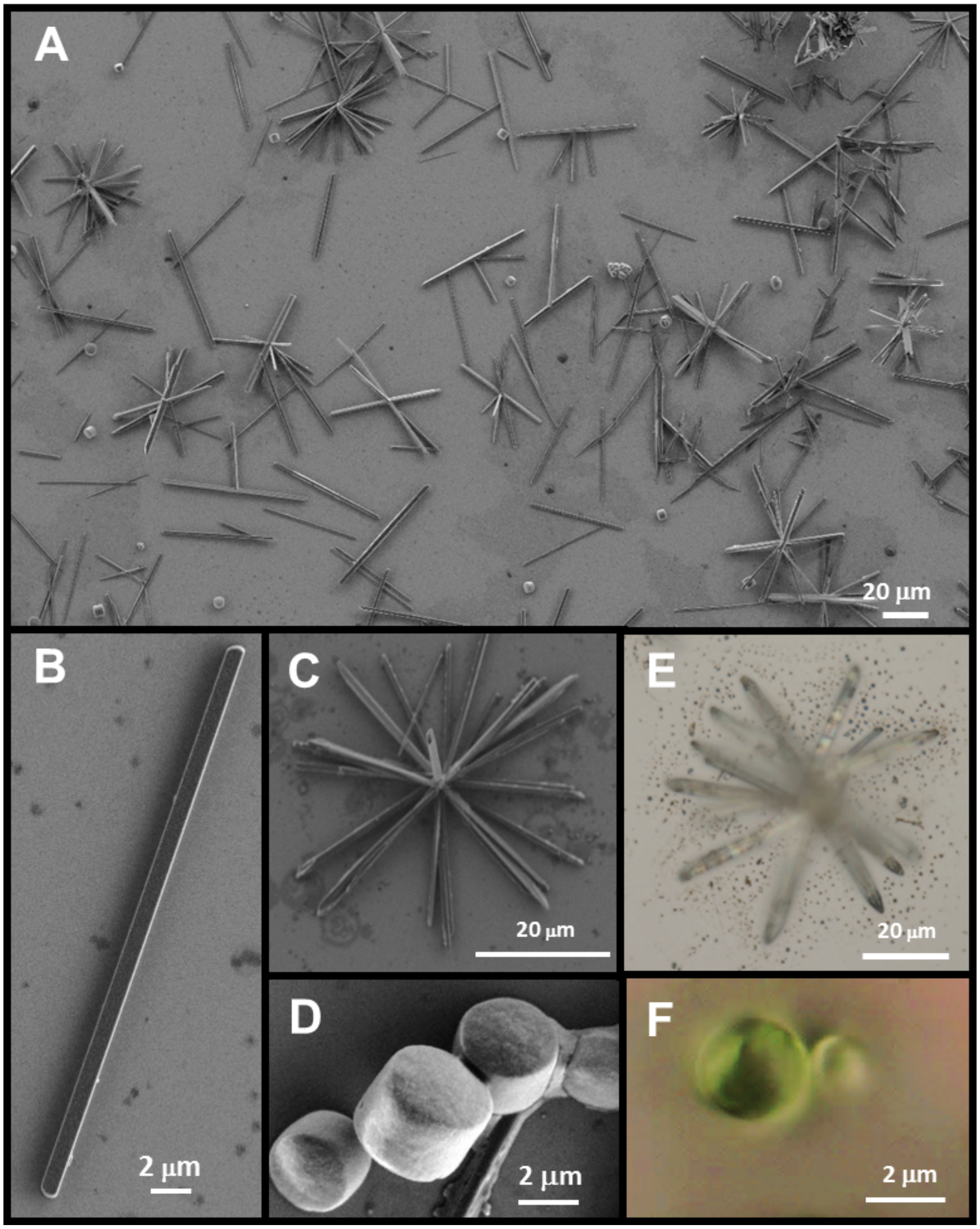

Supplementary Fig. 4. Crystal morphology after solvothermal reaction. (A) SEM image showing the structures forming after two days of solvothermal reaction. (B-D) zoom-in. (E, F) optical microscopy images.

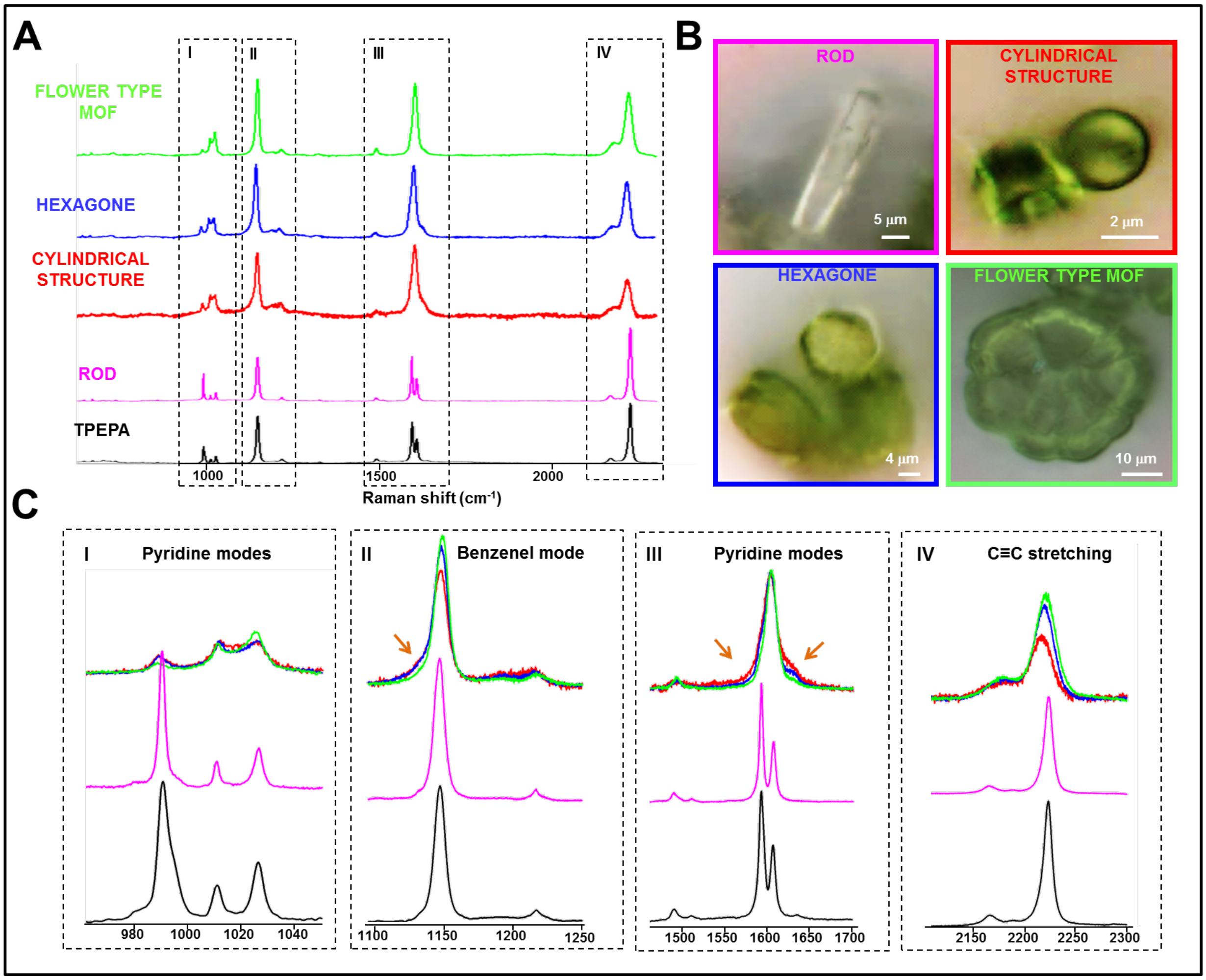

Supplementary Fig. 5. Crystal evolution by MicroRaman. (A) MicroRaman spectra of TPEPA (black), cylindrical structures after 2 days of solvothermal reaction (red) and a hexagon (blue), a rod (pink), a flower (green) structures after 2 days of solvothermal reaction followed by 11 hours (hexagon, rod) and 2 days (flower) of aging at room temperature. (B) Optical microscopy images of the structures analyzed by MicroRaman. (C) Zoom in of the MicroRaman spectra regions highlighted in A.

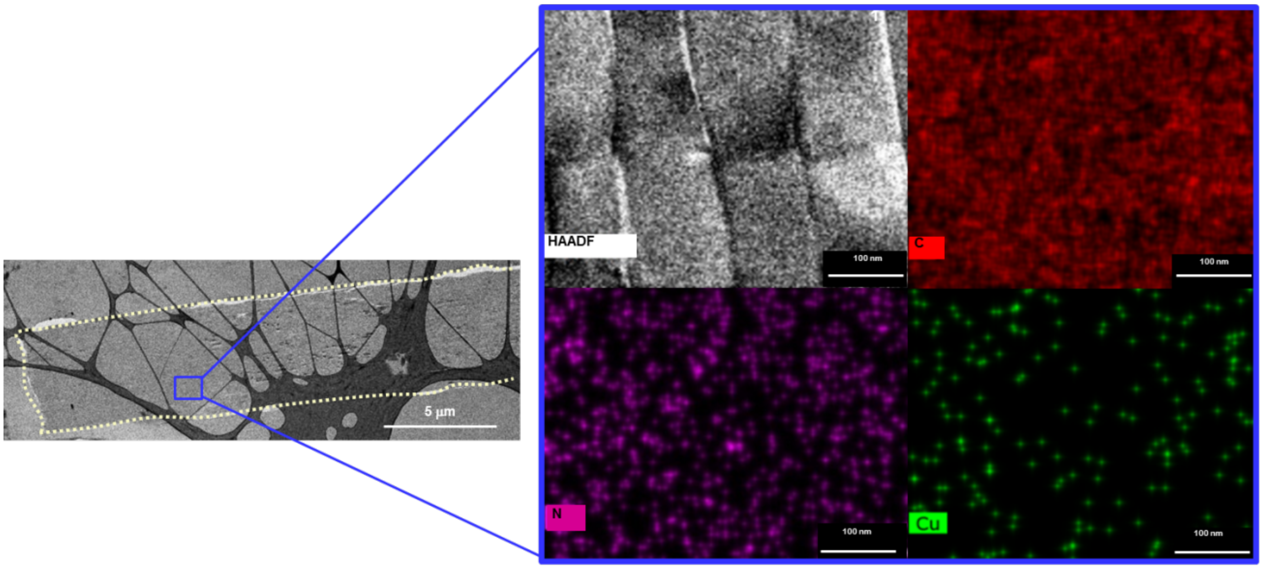

Supplementary Fig. 6. Rod-like crystal analysis by TEM. TEM images (left) of a microtome-assisted cut lamella of a rod structure after 2 days of solvothermal reaction followed by 1 day of aging at room temperature. The yellow dashed-lines highlight the profile of the rod; (right) annular dark-field image (HAADF) and elemental mapping of the region highlighted in blue in the TEM image. The striped texture, evident both in the TEM and HAADF images, is induced by the cutting.

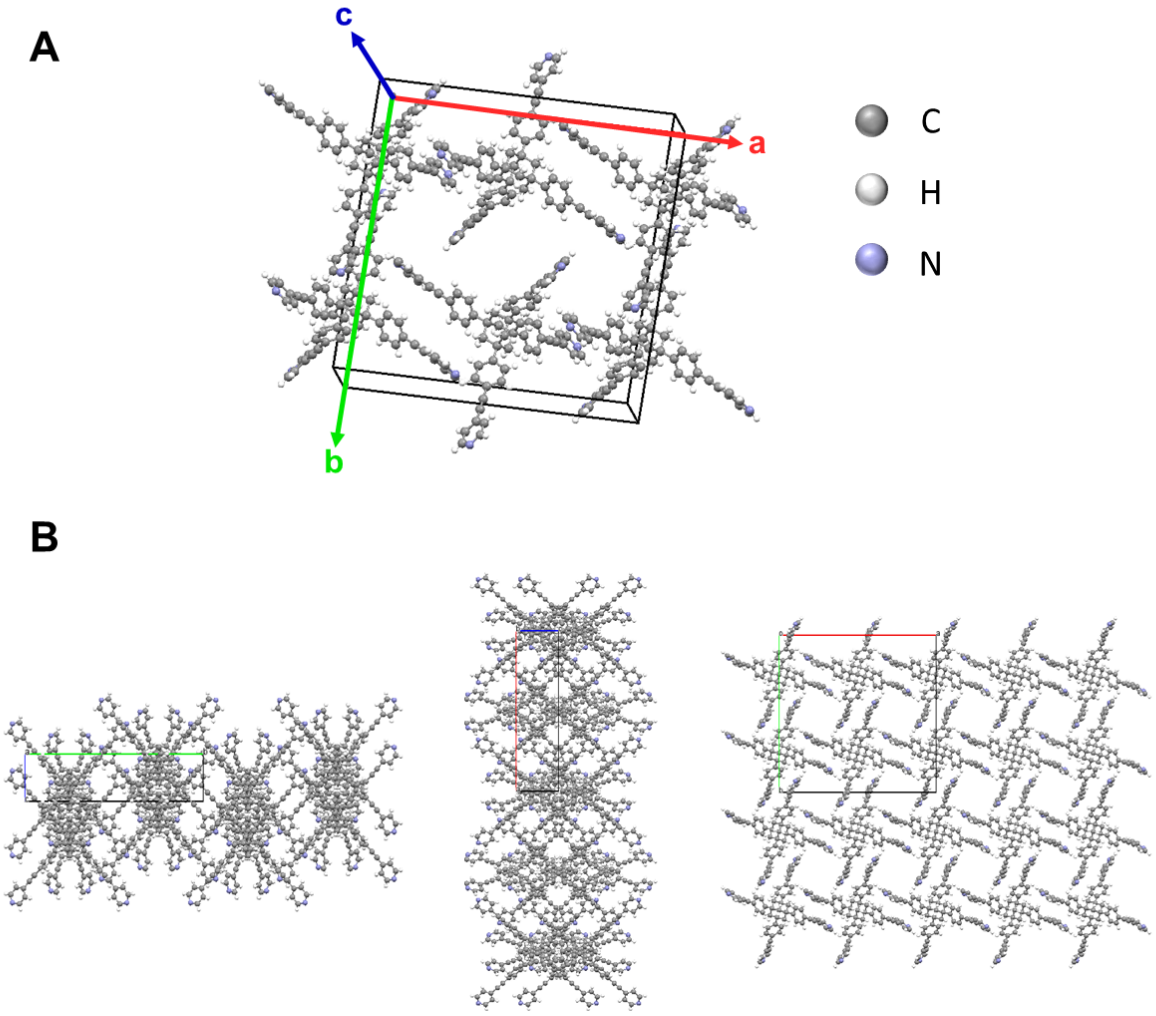

Supplementary Fig. 7. Rod-like crystal analysis by X-ray. Single crystal X-ray data of a rod-like structure consisting of TPEPA after 2 days of aging at room temperature: (A) unit cell; (B) view down the a (left), b (center) and c (right) axis. Space group $I4_1/a$, unit cell parameters: a = 26.7291(4) Å, b = 26.7291(4) Å, c = 7.05940(15) Å; $\alpha = 90°$, $\beta = 90°$, $\gamma = 90°$

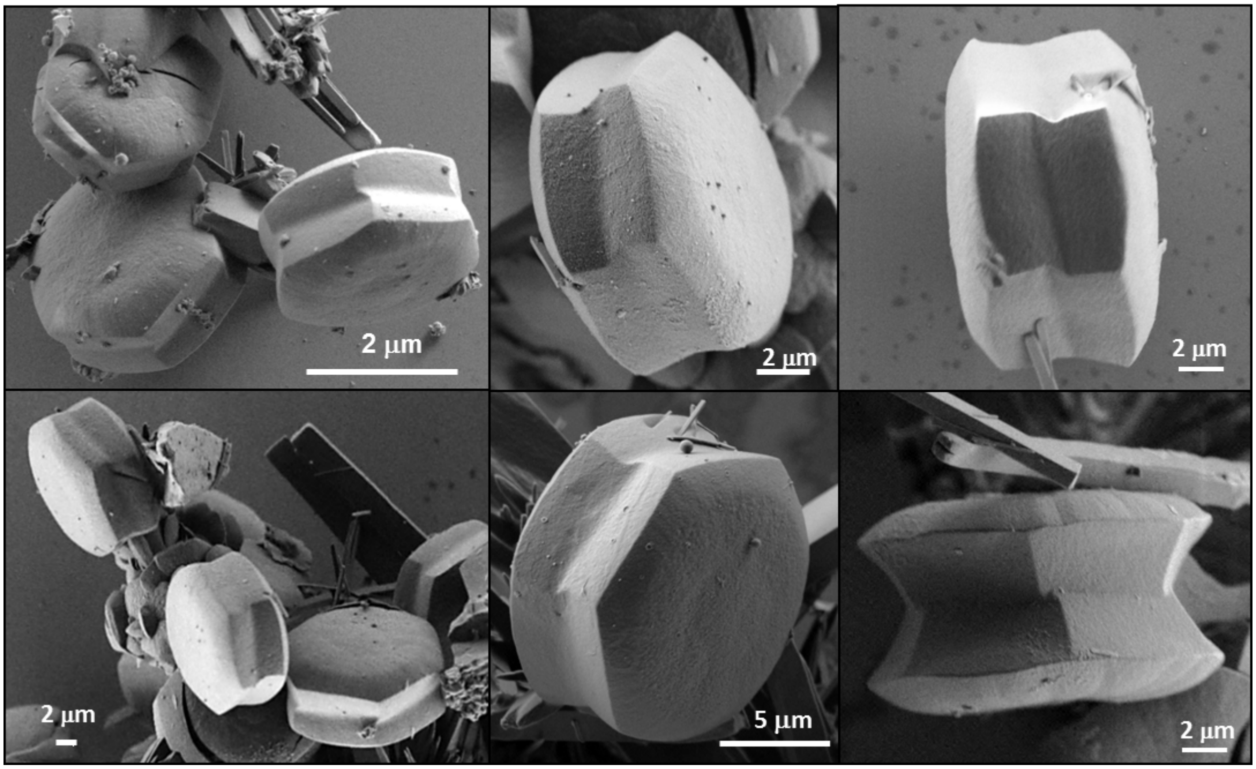

Supplementary Fig. 8. Structure morphology at 11 h of aging time. SEM images of the hexagonal structures after 2 days of solvothermal reaction followed by 11 hours of aging at room temperature.

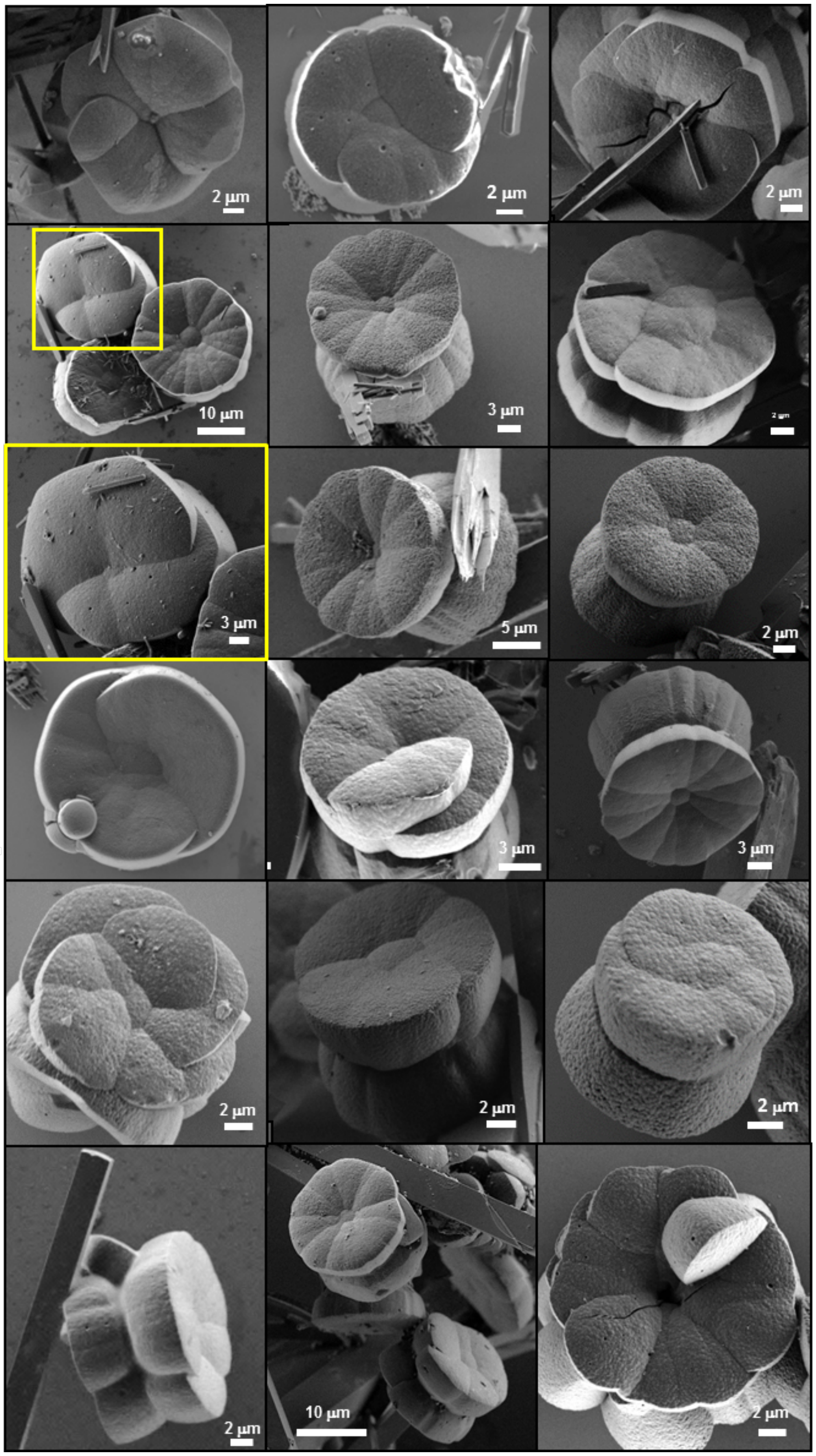

Supplementary Fig. 9. Structure morphology of intermediate yo-yo-like structures. SEM images of the yo-yo-like structures after 2 days of solvothermal reaction followed by 24 hours of aging at room temperature.

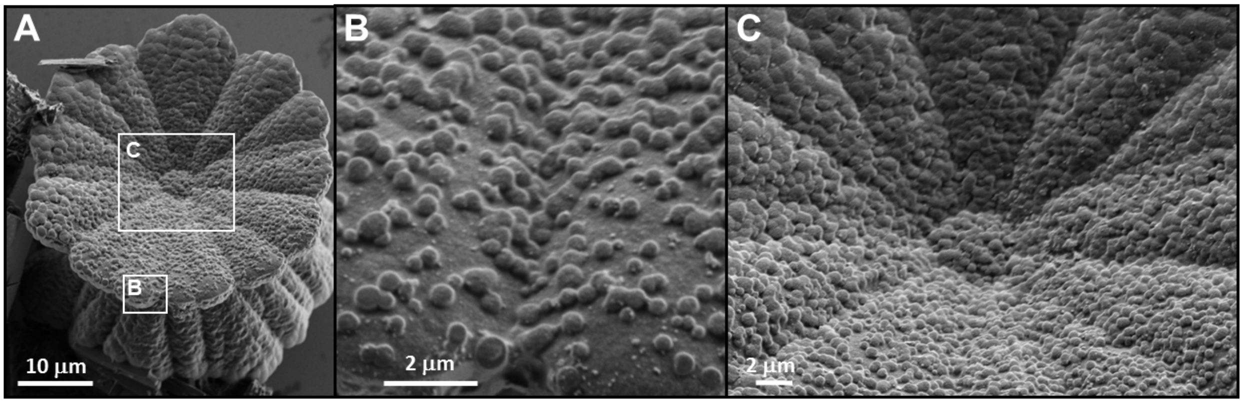

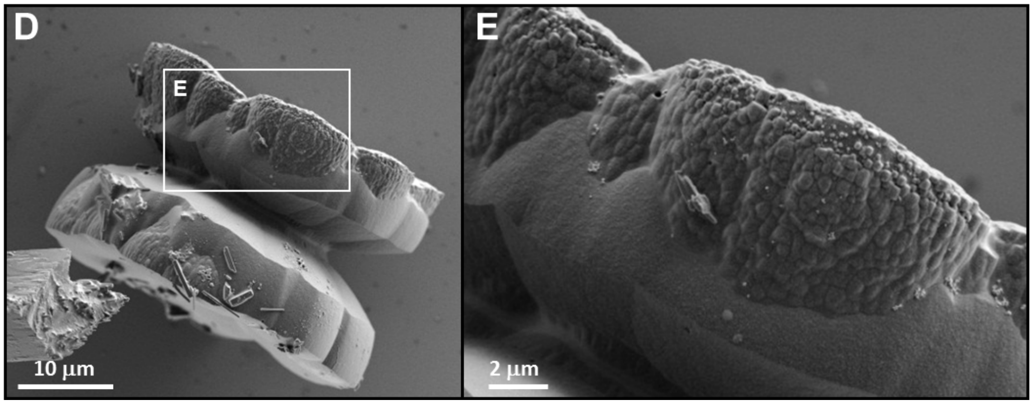

Supplementary Fig. 10. Birth and spread growth. (A-E) SEM images of the yo-yo-like structures after 2 days of solvothermal reaction followed by 2 days of aging at room temperature. The micrographs in B, C and E correspond to the regions marked by white squares in panels A and D, respectively.

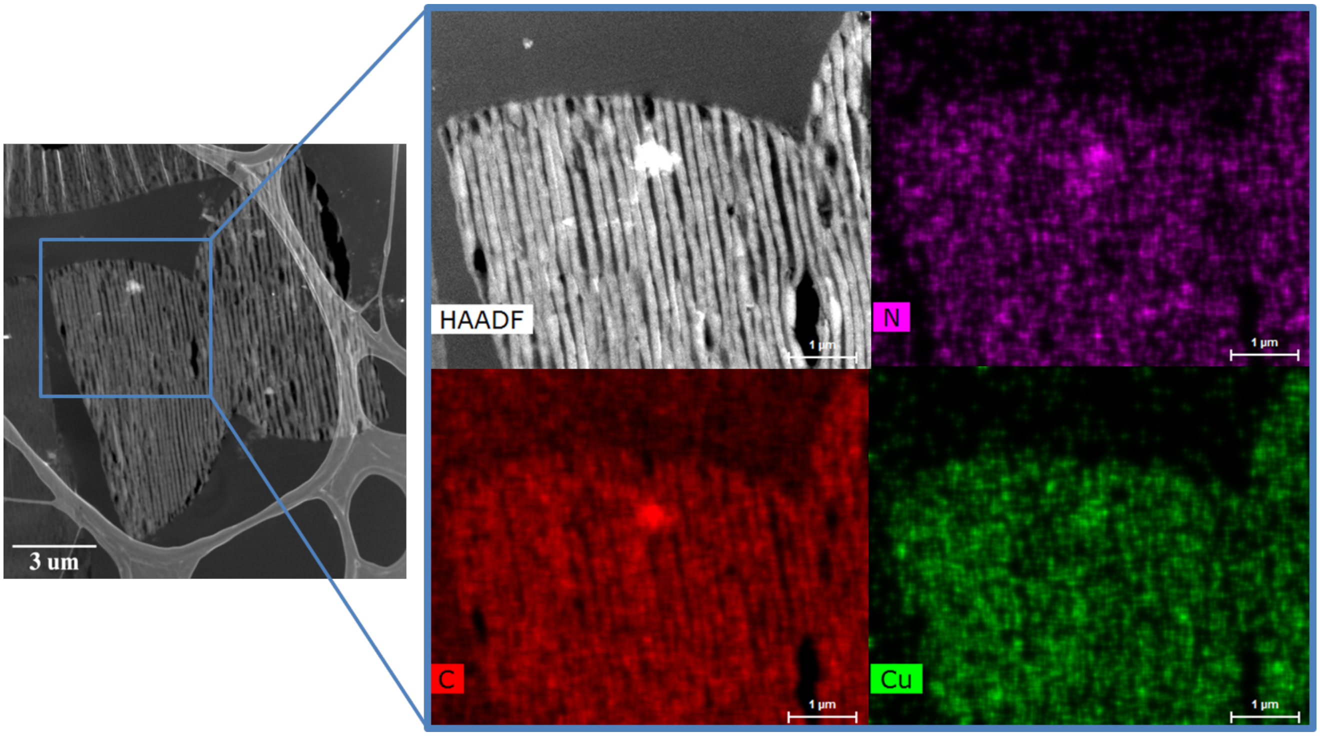

Supplementary Fig. 11. Yo-yo-like crystal analysis by TEM. TEM images (left) of a microtome-assisted cut lamella of a yo-yo-like crystal after 2 days of solvothermal reaction followed by a 1 day of aging at room temperature; (right) annular dark-field image (HAADF) and elemental mapping of the region highlighted in blue in the TEM image. The striped structure, evident both in the TEM and HAADF images, is induced by the cutting.

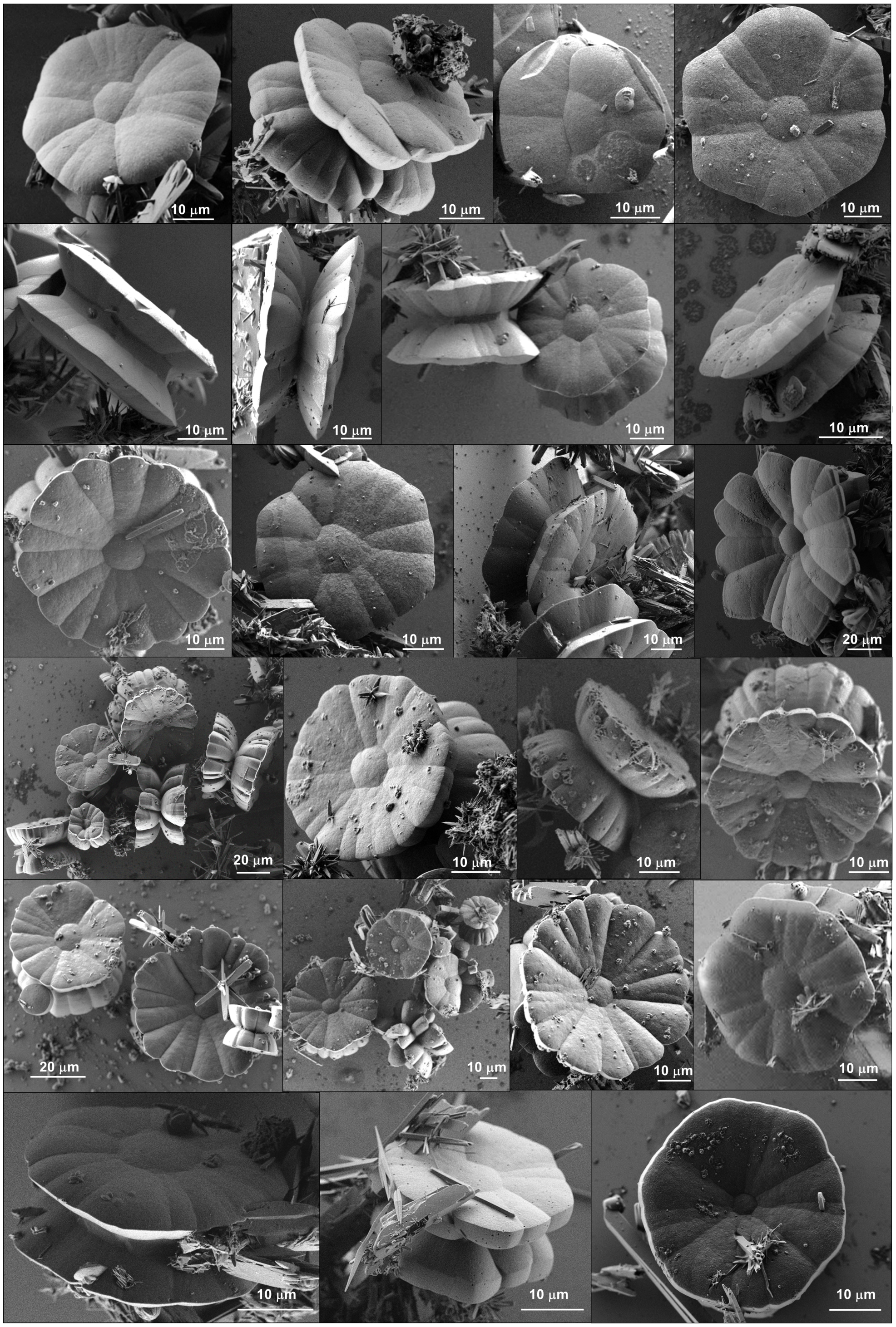

Supplementary Fig. 12. Yo-yo-like single crystals. SEM images of the yo-yo-like structures after 2 days of solvothermal reaction followed by 2 days of aging at room temperature.

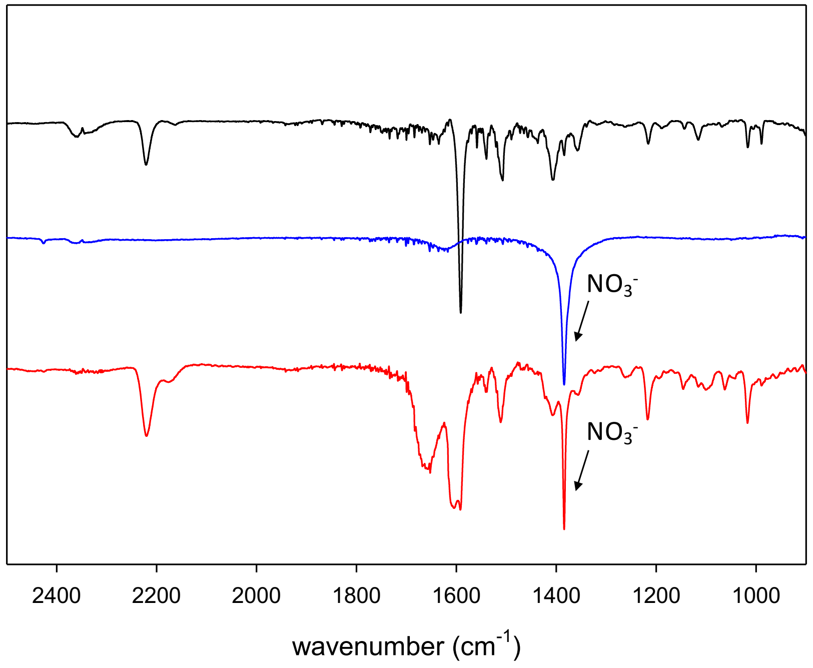

Supplementary Fig. 13. Bulk sample analysis by Infrared spectroscopy. Infrared spectra of **TPEPA** (black), $Cu(NO_3)_2{\cdot}H_2O$ (blue), sample after 2 days of ageing (red). KBr pallets were used to record these spectra.

Supplementary References

1. G. M. Sheldrick, Crystal structure refinement with SHELXL *Acta Crystallogr., Sect. A: Fund. Crystallogr.* **71**, 3-8 (2015)
2. S. Parsons, H. D. Flack, T. Wagner, Use of intensity quotients and differences in absolute structure refinement *Acta Cryst.* **B69**, 249-259 (2013)
