# Supplementary material for: Emergence of chirality and structural complexity in single crystals at the molecular and morphological levels
Source: Nat Commun. 2020 Jan 20;11:380. doi: 10.1038/s41467-019-13925-5 (PMC6971082; doi:10.1038/s41467-019-13925-5)
Supplement: Supplementary file 2 — Description of Additional Supplementary Files [file 41467_2019_13925_MOESM2_ESM.pdf]

Description of Additional Supplementary Files

File name: Supplementary Movie 1
Description: microCT volume rendering of a yo-yo-like crystal, a snapshot is shown in Fig. 3A.

File name: Supplementary Movie 2
Description: microCT volume rendering after performing segmentation of a yo-yolike crystal, a snapshot is shown in Fig. 3B.

File name: Supplementary Movie 3
Description: Propeller-type coordination of the yo-yo-like crystal shown in orange in the main text (CCDC 1910232).

File name: Supplementary Movie 4
Description: Propeller-type coordination center of the yo-yo-like crystal shown in green in the main text (CCDC 1910233).

File name: Supplementary Movie 5
Description: Outer and inner helicoids composing the hexagonal channel in the crystal structure of the yo-yo-like crystals

File name: Supplementary Movie 6
Description: Outer helicoids of the hexagonal channel in the crystal structure of the yo-yo-like crystals.

File name: Supplementary Movie 7
Description: Inner helicoids of the hexagonal channel in the crystal structure of the yo-yo-like crystals.

File name: Supplementary Movie 8
Description: Helicoids composing the wall of the triangular channels in the crystal structure of the yo-yo-like crystals.
